# Regulation of the cecal microbiota community and the fatty liver deposition by the addition of brewers’ spent grain to feed of Landes geese

**DOI:** 10.3389/fmicb.2022.970563

**Published:** 2022-09-20

**Authors:** Ping Xu, Yuxuan Hong, Pinpin Chen, Xu Wang, Shijie Li, Jie Wang, Fancong Meng, Zutao Zhou, Deshi Shi, Zili Li, Shengbo Cao, Yuncai Xiao

**Affiliations:** ^1^College of Veterinary Medicine, Huazhong Agricultural University, Wuhan, China; ^2^State Key Laboratory of Agricultural Microbiology, Huazhong Agricultural University, Wuhan, China; ^3^Key Laboratory of Preventive Veterinary Medicine in Hubei Province, Huazhong Agricultural University, Wuhan, China

**Keywords:** brewers’ spent grain, Landes goose, fatty liver, cecal microbiota community, differentially expressed gene, transcriptomics

## Abstract

The effects of brewers’ spent grain (BSG) diets on the fatty liver deposition and the cecal microbial community were investigated in a total of 320 healthy 5-day-old Landes geese. These geese were randomly and evenly divided into 4 groups each containing 8 replicates and 10 geese per replicate. These four groups of geese were fed from the rearing stage (days 5–60) to the overfeeding stage (days 61–90). The Landes geese in group C (control) were fed with basal diet (days 5–90); group B fed first with basal diet in the rearing stage and then basal diet + 4% BSG in the overfeeding stage; group F first with basal diet + 4% BSG during the rearing stage and then basal diet in the overfeeding stage; and group W with basal diet + 4% BSG (days 5–90). The results showed that during the rearing stage, the body weight (BW) and the average daily gain (ADG) of Landes geese were significantly increased in groups F and W, while during the overfeeding stage, the liver weights of groups W and B were significantly higher than that of group C. The taxonomic structure of the intestinal microbiota revealed that during the overfeeding period, the relative abundance of *Bacteroides* in group W was increased compared to group C, while the relative abundances of *Escherichia–Shigella* and *prevotellaceae_Ga6A1_group* were decreased. Results of the transcriptomics analysis showed that addition of BSG to Landes geese diets altered the expression of genes involved in PI3K-Akt signaling pathway and sphingolipid metabolism in the liver. Our study provided novel experimental evidence based on the cecal microbiota to support the application of BSG in the regulation of fatty liver deposition by modulating the gut microbiota in Landes geese.

## Graphical Abstract

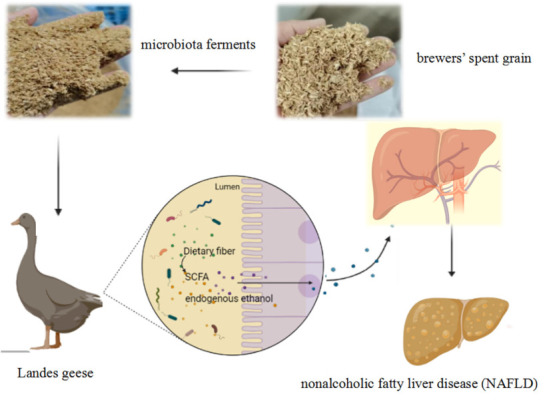

## Introduction

It is well-known that in the natural environment, some wild waterfowl (e.g., geese and ducks) would eat a large amount of food in a short period of time to deposit excess fat in the liver to form fatty liver prior to migration in order to meet the energy reserve needs of long-distance travel (Hermier et al., 1991). Studies showed that different from the human fatty liver, geese have shown a strong ability to store fat in their livers with the functional integrity of the liver cells preserved without the formation of fibrosis or hepatic necrosis (Lu et al., 2015). Therefore, geese are used extensively worldwide in the production of luxurious food based on their fatty liver products, e.g., foie gras. The foie gras is generally delicious and delicate in texture, favored by a large number of consumers worldwide, with an extremely large and increasing demand in the international market (Mozduri et al., 2021). In particular, as a world-renowned breed dedicated to fatty livers, the Landes geese are very popular and highly adaptable to their environment (Hermier et al., 1999; Mourot et al., 2000).

The brewers’ spent grain (BSG) is the main by-product generated during the beer production (Mussatto, 2014), mainly composed of malt husk, insoluble protein, hemicellulose, fat, and a small amount of undecomposed starch (Cooray et al., 2017; Rachwał et al., 2020). Specifically, both compounds β-glucans and arabinoxylan detected in BSG are consumed by animals to help enhance the activities of beneficial microbiota (Lao et al., 2020). Studied showed that sun-dried BSG added to the diets for pigs at a dose of 17–25% improved the production margins (Amoah et al., 2017), while it was demonstrated that replacing 5% soybean meal with fermented BSG in the diet of Wanxi white geese increased both the feed intake and body weight of meat geese (Yang et al., 2020). Furthermore, studies showed that BSG contained microorganisms capable of producing endogenous ethanol (Bonifácio-Lopes et al., 2020), which promoted the accumulation of fat in the liver (Baraona and Lieber, 1979). These results strongly suggested the significant potential of BSG to promote liver fat deposition. However, the molecular mechanism underlying the liver fat deposition promoted by BSG is still unclear.

As the largest metabolic organ in body, the liver plays important roles in regulating the glucose and lipid metabolisms, while the intestine is the main place where the nutrient digestive absorption takes place and the gut microbiota colonizes, playing an important role in the growth and metabolism of the hosts (Safari and Gerard, 2019). The “gut-liver axis” refers to the bidirectional relationships among intestinal microbiome, intestine, and liver, attracting significant attention due to its involvement with intestinal microbiota in the occurrence and development of non-alcoholic fatty liver disease (NAFLD) (Bajaj and Hylemon, 2018). Studies have shown that overfeeding could cause significant variations in gut physiology and gut microbiota, thereby regulating the formation of fatty livers in geese (Knudsen et al., 2021). To date, the studies on the gut microbiome of Landes geese are sparse, while the investigations exploring the effects of BSG diets on the improvement of the liver production in Landes geese through the “gut-liver axis” are still lacking. Therefore, in this study, we performed the analyses of the intestinal physiology, intestinal microbiota, and liver transcriptomics of Landes geese to investigate the beneficial effects of BSG on the varied regulatory patterns of lipid deposition in Landes geese. The overall physiological and developmental benefits in the Landes geese generated by the addition of BSG in the feed were characterized based on the taxonomic composition of the intestinal microbiota before and after the overfeeding stage as well as changes in their body weight, liver weight, blood biochemical indices, and nutrient composition in the liver. The protective mechanism of the nutritional fatty liver in the Landes geese was further explored with the potential solutions to the problems related to fatty liver in animals. Our study provided novel experimental evidence to support the further investigations and applications of BSG in the promotion of fatty liver development in Landes geese.

It is noted that the overfeeding method is currently a controversial issue in the field. Interestingly, with the continuous improvement of modern overfeeding technology and equipment, the overfeeding method of geese is becoming more and more mature and acceptable. In particular, during the entire feeding process, we followed closely the provisions of the Chinese Code of Practice for Testing the Performance of Geese with Fatty Liver (NT/T 3184-2018). Furthermore, the Landes geese are physiologically characterized by fatty deposits in the liver. It is expected that with the discovery of the relationships between liver fat deposition and intestinal microbiota through overfeeding, in the near future, it would be possible to directly achieve the liver fat deposition in geese by regular feeding of certain types of feed additions (e.g., BSG) without overfeeding the geese. This achievement would be of significant importance for fatty liver production.

## Materials and methods

### Laboratory animals

This study was performed by following strictly the Guide for the Care and Use of Laboratory Animals Monitoring Committee of Hubei Province, China. The experimental protocols were approved by the Committee on the Ethics of Animal Experiments of the College of Veterinary Medicine, Huazhong Agricultural University (NO. HZAUGE–2020–0001).

A total of 320 healthy 5-day-old Landes geese were randomly and evenly divided into 4 groups with each group containing 8 replicates and 10 geese per replicate. The geese were kept and fed in the brooding room for a total of 4 days prior to the start of the experiments. The Landes geese in group C (control) were fed with basal diet throughout the entire experiment in both the rearing stage (days 5–60) and the overfeeding stage (days 61–90); group B first with basal diet in the rearing stage and then with basal diet + 4% BSG in the overfeeding stage; group F first with basal diet + 4% BSG in the rearing stage and then basal diet in the overfeeding stage; and group W with basal diet + 4% BSG for the entire experiment (days 5–90). No animals were treated with antibiotics during the entire experiments. The Landes geese at different developmental stages were fed with different basal feeds with varied formula of nutritional compositions (Supplementary Tables 1–3).

The animal experiments were carried out at the Huaren Modern Farm (Anhui, China) according to the code of practice for performance testing of fatty liver goose in China (NT/T 3184–2018). The entire experimental procedures for four groups of Landes geese (i.e., C, B, F, and W) were divided into two stages. First, in the rearing stage (days 5–60), the temperature in the goose houses where the Landes geese were kept and fed was maintained at ∼33°C until the geese were 7 days old; then, the temperature was gradually decreased to and maintained at 23°C. It was noted that in days 45–60, reasonable feed consumption control was applied in order to prevent rapid excess body weight in the geese. The goose houses were disinfected with both potassium permanganate and formalin before the experiments started. Second, in the overfeeding stage (days 61–90), the temperature in the goose houses was maintained at ∼15°C. After the pre-feeding (days 61–67) in the overfeeding stage, the force feeding frequency and ration were gradually increased, i.e., force feeding for 2–3 times/day (days 68 and 69) with 140 g/time, 3–4 times/day (days 70–74) with 220 g/time, and 5–6 times/day (days 75–90) with 350 g/time. The feeds were softened with water during the overfeeding stage. On days 5, 15, 30, 45, 60, and 90, the geese and feeds were weighed to calculate the average daily gain (ADG) in body weight (BW) in each of the four groups of geese (n = 80) based on the following equation: ADG = (final body weight − initial body weight)/days of experiment.

### Brewers’ spent grain

As the residue of barley malt extracted during the wort production process, the BSG was supplied by the Hubei Huada Real Technology Co., Ltd., Wuhan, China. The nutritional compositions of the BSG were shown in Table 1.

**TABLE 1:** The nutritional compositions of brewers’ spent grain (BSG).

Ingredient	Percentage in the total weight
Water content	36.97%
pH	4.23
Total acid	2.08%
Crude protein	13.26%
Crude fiber	15.70%
Crude fat	6.30%
Crude ash	3.70%
Acid-soluble protein (as a percentage of the crude protein)	25.73%

### Sample collection

A total of eight geese were sampled from each of the four groups of Landes geese on days 60 and 90 after 12 h of fasting with the pen number and weight of each individual goose recorded before euthanization. Blood samples (∼5 mL for each goose) were collected from the wing veins using the vacuum blood collection tubes and centrifuged for 10 min at 3000 rpm and 4°C to obtain the serum sample. The geese were euthanized with the liver tissues (on day 90) collected immediately, snap-frozen in liquid nitrogen, and stored at −80°C for further transcriptomics analysis. The duodenal, jejunal, and ileal tissues were collected and immediately fixed in 4% paraformaldehyde (Biosharp Co., Ltd., Hefei, China) for subsequent morphological analysis.

### Serum analysis

The contents of a group of nine biochemical indices, including alanine aminotransferase (ALT), aspartate aminotransferase (AST), alkaline phosphatase (AKP), acid phosphatase (ACP), glucose (GLU), triglyceride (TG), cholesterol (CHO), high density liptein cholesterol (HDL-C), and low density liptein cholesterol (LDL-C), were measured by an automatic biochemical analyzer (BK-280, Shandong Blobase Biotechnology Co., Ltd., Shandong, China), while the contents of another biochemical index, i.e., the very low-density lipoprotein (VLDL-C), were measured using ELISA kits (Wuhan Meimian Biotechnology Co., Ltd., Wuhan, China). All experiments were performed with eight biological replicates in strict accordance with the protocols and instructions recommended by the manufacturers.

### Compositions of free amino acids in livers of Landes geese

The contents of amino acids in livers of Landes geese on day 90 were determined using an amino acid analyzer (L-8900, Hitachi, Japan). A total of ∼100 mg liver samples were dissolved in water with methanol (1:1) for 30 min at 4°C and then centrifuged for 10 min at 10,000 × g and 4°C. The supernatant was filtered through the glass wool and stored at −80°C for further analysis (Dong et al., 2020).

### Compositions of fatty acids in livers of Landes geese

The fatty acid compositions in livers of Landes geese on day 90 were measured using the gas chromatography (GC) based on the methods described previously (Li et al., 2015) using the gas chromatographer (Trace1310 ISQ, ThermoFisher, Waltham, CA, United States). The concentrations of individual fatty acids were quantified based on the peak area and presented as a percentage of the contents of the total fatty acids.

### Gut microorganism analysis

#### Sample collection and DNA extraction and sequencing

The total genomic DNA of the microbial community was extracted from the cecal contents using the E.Z.N.A.^®^ Soil DNA Kit (Omega Bio-tek, Norcross, GA, United States) by following the procedures recommended by the manufacturers. DNA quality was evaluated on 1% agarose gel with the DNA concentration and purity determined by the NanoDrop 2000 UV-vis spectrophotometer (Thermo Scientific, Wilmington, DE, United States). Then, the forward primer 338F (5′-ACTCCTACGGGAGGCAGCAG-3′) and the reverse primer 806R (5′-GGACTACHVGGGTWTCTAAT-3′) were used to amplify the hypervariable region (i.e., V3-V4) of the bacterial 16S rRNA gene on the PCR thermocycler (ABI GeneAmpR^®^ 9700, Foster City, CA, United States) with the following procedures: denaturation for 3 min at 95°C, followed by a total of 27 cycles of denaturation for 30 s at 95°C, annealing for 30 s at 55°C, and extension for 45 s at 72°C, ended by the final extension for 10 min at 72°C, and kept at 4°C. The chemical mixture of PCR contained 4 μL 5 × TransStart FastPfu buffer, 2 μL 2.5 mM dNTPs, 0.8 μL forward primer (5 μM) and reverse primer (5 μM), 0.4 μL TransStart FastPfu DNA Polymerase, and 10 ng template DNA, with the final volume adjusted to 20 μL using ddH_2_O. Each PCR analysis was repeated with three biological replicates. The PCR products were collected using 2% agarose gel and then purified using the AxyPrep DNA Gel Extraction Kit (Axygen Biosciences, Union City, CA, United States) by following the manufacturer’s instructions. The concentrations of the purified PCR products were determined using the Quantus™ Fluorometer (Promega, United States). The paired-end sequencing (2 × 300 bp) of the purified amplicons pooled in equimolar was performed on an Illumina MiSeq platform (Illumina, San Diego, CA, United States) based on the standard procedures recommended by the Majorbio Bio-Pharm Technology Co., Ltd. (Shanghai, China).

#### Sequencing data analysis

The paired-end reads of the transcriptomics analysis were processed using FLASH version 1.2.11 to generate the splicing sequences, i.e., the raw tags (Derakhshani et al., 2016). Further processing of raw reads was performed based on the quality control protocols of QIIME version 1.9.1 (Caporaso et al., 2010). The effective tags were clustered into the operational taxonomic units (OTUs) based on 97% identity with the representative sequences of the OTUs determined and annotated using the Uparse version 7.0.1090^1^. The taxonomy of each OTU representative sequence was determined by the RDP Classifier^2^ based on the 16S rRNA database (Zhbannikov and Foster, 2015). The alpha diversity indices (i.e., Chao1, Shannon, and Simpson) were calculated based on the rarefaction analysis using Mothur v.1.30.2, while the relative abundance analyses at the phylum and genus levels were performed using the R software (version 3.3.1).

### Transcriptomics analysis of liver in Landes geese

#### RNA extraction and sequencing

The total RNA of each liver sample was extracted using the Ultrapure RNA Kit (CW0581M, CoWin Biosciences, Beijing, China) according to the manufacturer’s instructions. The RNA samples meeting the quality requirements, i.e., bright and clear bands of the target RNA based on electrophoresis gel, no diffusion area in the swimming lane, no protein and DNA contaminations, with the RNA integrity number (RIN) close to 10, the 28S/18S ratio greater than or equal to 1.5, 1.8 < OD_260/280_ < 2.2, and OD_260/230_ ≥ 2.0, were used to construct the RNA-Seq transcriptomic library (Majorbio Co., Shanghai, China).

#### Sequencing data analysis

The differentially expressed genes (DEGs) between the four different groups of liver samples of Landes geese on day 90 were identified based on fold change > 2 or < –2 and *Q* value ≤ 0.05 using DESeq2 (1.24.0)^3^. The expression level of each transcript was determined by the transcripts per million reads (TPM) method, while the gene abundances were evaluated based on RSEM^4^. Annotation and enrichment analyses of the DEGs based on the Gene Ontology (GO^5^) and the Kyoto Encyclopedia of Genes and Genomes (KEGG^6^) databases were performed by Goatools^7^ and KOBAS^8^, respectively, with the Bonferroni-corrected *P*-value ≤ 0.05 compared with the entire transcriptome background.

In order to verify the molecular patterns revealed by the transcriptomic analysis, a total of six genes were randomly selected to perform the quantitative real-time PCR (qRT-PCR) analysis. The RNA sample (1 μg) was reverse-transcribed into cDNA using the PrimeScript™ RT Reagent Kit with gDNA Eraser (Vazyme, Nanjing, China) by following the manufacturer’s protocols. Then, the cDNA was diluted 10-fold and mixing with ChamQ Universal SYBR qPCR Master Mix (Q711-02/03, Vazyme Biotech Co., Ltd., Nanjing, China) and specific primers, used for qRT-PCR analyses using the signal detection protocols provide by the manufacturers (Bio-Rad CFX96TM System, TaKaRa, Dalian, China). Each qRT-PCR experiment was repeated with three technical replicates using the house-keeping gene *β-Actin* as the endogenous control for the normalization of the expression of each gene. Primers used for qRT-PCR were shown in Supplementary Table 4. Data were analyzed using GraphPad Prism v 8.3.0 (GraphPad, Inc., La Jolla, CA, United States).

### Statistical analysis

The significant differences between groups were analyzed by one-way analysis of variance (ANOVA) and Fisher’s least significant difference (LSD) tests using the SPSS statistical software version 26.0 (SPSS, Inc., Chicago, IL, United States). Graphs were generated using GraphPad Prism 8.3. (GraphPad, Inc., La Jolla, CA, United States). The data were shown as the mean ± standard error of the mean (SEM) with the significance levels set at *P* < 0.05 (*) and *P* < 0.01 (^**^), respectively.

## Results

### Effect of brewers’ spent grain on the growth performance of Landes geese

The results of growth performance in the Landes geese showed that the initial BWs were not significantly different (*P* > 0.05) among the 4 groups of Landes geese (Table 2). In the rearing stage (days 5–60), the geese in both groups F and W fed with diets supplemented with BSG showed higher BW and ADG than those of group C, with BW extremely significantly different at 15, 30, and 45 days (*P* < 0.01) and ADG extremely significantly different during 5–15 and 16–30 days (*P* < 0.01). During the overfeeding stage (days 61–90), the BSG was added to the diet of group B, but the BW and ADG were not changed significantly compared with group C. In 90 days, the average liver weight/body weight ratio of the geese in group W was increased by 21.36% compared with group C, while the liver weight/body weight ratio of group B also showed an increasing trend compared with groups C and F (*P* > 0.05) (Table 2). Overall, these results showed that addition of BSG during the rearing stage effectively increased the BW and ADG of Landes geese, while no significant difference was observed in BW with the addition of BSG during the overfeeding stage. Therefore, the addition of BSG during the overfeeding stage was beneficial to the liver fat deposition of the Landes geese.

**TABLE 2:** Effects of brewers’ spent grain (BSG) on the growth performance in four groups of Landes geese (i.e., groups C, B, F, and W).

Growth		Group C	Group B	Group F	Group W	*P*-value
BW (g)	5 days	218.7 ± 1.3	218.1 ± 1.3	218.1 ± 1.9	217.5 ± 1.3	0.952
	15 days	703.0 ± 3.7^b^	711.0 ± 3.9^b^	726.2 ± 2.7^a^	733.5 ± 3.4^a^	< 0.001
	30 days	1913.7 ± 43.6^b^	1916.1 ± 36.9^b^	2080.0 ± 39.3^a^	2087.5 ± 47.1^a^	0.005
	45 days	3385.6 ± 35.6^b^	3383.1 ± 48.2^b^	3698.1 ± 45.4^a^	3603.8 ± 59.2^a^	0.001
	60 days	4531.3 ± 51.5	4522.5 ± 69.9	4678.9 ± 48.2	4671.9 ± 81.8	0.174
	90 days	8066.7 ± 98.9	8300.0 ± 134.2	8480.0 ± 135.6	8400.0 ± 219.1	0.299
LW (g)	90 days	946.7 ± 46.5^b^	1089.2 ± 67.3^ab^	943.3 ± 61.9^b^	1192.5 ± 36.3^a^	0.010
LBR	90 days	11.7 ± 0.5	13.1 ± 0.8	11.6 ± 0.8	14.2 ± 0.7	0.053
ADG (g/d)	5–15 days	48.5 ± 0.2^b^	48.9 ± 0.3^b^	51.4 ± 0.3^a^	50.8 ± 0.2^a^	< 0.001
	16–30 days	80.6 ± 2.8^b^	80.5 ± 2.5^b^	90.4 ± 3.2^a^	90.2 ± 2.6^a^	0.015
	31–45 days	98.1 ± 1.0	97.8 ± 4.6	101.0 ± 4.7	101.3 ± 2.7	0.852
	46–60 days	76.4 ± 1.1	75.9 ± 4.5	71.3 ± 6.1	71.9 ± 4.2	0.776
	61–90 days	117.3 ± 1.9	125.7 ± 3.5	124.7 ± 6.3	124.3 ± 2.5	0.492

Data are expressed as mean ± standard error of the mean (SEM) (n = 80 geese per group). ^a,b^Means within the same row with different superscripts differ. BW, body weight; LW, liver weight; LBR, liver weight/body weight ratio; ADG, average daily gain. Group C, control group; Group B, added with 4% BSG in the overfeeding stage (days 61–90); Group F, added with 4% BSG in the rearing stage (days 5–60); Group W, added with 4% BSG in the all stage (days 5–90).

### Effect of brewers’ spent grain on the serum biochemical indices of Landes geese

The results of the effects of BSG on the serum biochemical indices of Landes geese were shown in Table 3. In 60 days, compared with group C, the contents of GLU were extremely significantly reduced in both groups F and W (*P* < 0.01), while the contents of HDL-C were significantly decreased (*P* < 0.05) and the contents of LDL-C (*P* < 0.01) and VLDL-C (*P* > 0.05) showed an increasing. In 90 days, compared with group C, the contents of both ALT and AST in the three experimental groups (i.e., groups B, F, and W) were reduced, while the contents of TP in all three experimental groups were increased. No significant difference was observed in the contents of TG and CHO among the four groups of Landes geese (*P* > 0.05). Compared with group C, the contents of three types of lipoproteins (i.e., HDL-C, LDL-C, and VLDL-C) in group W were increased by 16.60% (*P* < 0.05), 25.00% (*P* > 0.05), and 34.96% (*P* < 0.01), respectively.

**TABLE 3:** Effects of brewers’ spent grain (BSG) on the serum biochemical indices in the four groups of Landes geese (i.e., groups C, B, F, and W) in 60 and 90 days.

Biochemical index	Group C	Group B	Group F	Group W	*P*-value
**Day 60**					
ALT (U/L)	23.1 ± 0.8	22.3 ± 0.7	23.2 ± 0.4	22.3 ± 1.4	0.834
AST (U/L)	62.4 ± 1.4	62.8 ± 1.9	58.9 ± 1.5	58.7 ± 1.3	0.139
AKP (U/100 mL)	61.8 ± 0.8	62.5 ± 0.8	61.5 ± 0.6	61.9 ± 0.5	0.749
ACP (U/100 mL)	20.9 ± 0.7	20.3 ± 0.9	19.7 ± 0.3	19.2 ± 0.9	0.434
GLU (mmol/L)	11.6 ± 0.3^a^	11.4 ± 0.3^a^	10.3 ± 0.3^b^	10.4 ± 0.3^b^	0.005
TG (mmol/L)	1.1 ± 0.1	1.1 ± 0.1	1.1 ± 0.1	1.0 ± 0.1	0.638
CHO (mmol/L)	4.6 ± 0.3	4.6 ± 0.2	4.4 ± 0.2	4.3 ± 0.1	0.158
HDL-C (mmol/L)	2.6 ± 0.2^a^	2.6 ± 0.2^a^	2.4 ± 0.1^b^	2.5 ± 0.1^b^	0.031
LDL-C (mmol/L)	1.2 ± 0.1^b^	1.1 ± 0.1^b^	1.3 ± 0.1^a^	1.3 ± 0.1^a^	0.008
VLDL-C (nmol/L)	291.7 ± 8.8	293.7 ± 26.6	320.6 ± 24.6	343.6 ± 22.7	0.323
**Day 90**					
ALT (U/L)	85.3 ± 1.5^a^	68.1 ± 4.4^b^	55.9 ± 3.7^b^	76.7 ± 3.9^a^	< 0.001
AST (U/L)	189.4 ± 12.0^a^	178.2 ± 13.1^a^	184.1 ± 15.1^a^	138.3 ± 5.5^b^	0.041
AKP (U/100 mL)	191.4 ± 11.2^a^	177.9 ± 17.3^a^	195.1 ± 7.3^a^	138.8 ± 7.3^b^	0.018
ACP (U/100 mL)	25.1 ± 1.6^b^	21.1 ± 1.7^b^	24.2 ± 2.1^b^	36.5 ± 1.2^a^	< 0.001
GLU (mmol/L)	16.4 ± 1.2^ab^	14.7 ± 0.5^b^	18.7 ± 1.5^a^	13.6 ± 0.6^b^	0.017
TG (mmol/L)	8.7 ± 0.8	6.7 ± 0.7	8.2 ± 0.5	6.8 ± 0.7	0.147
CHO (mmol/L)	7.2 ± 0.6	7.0 ± 0.3	6.4 ± 0.2	6.9 ± 0.4	0.478
HDL-C (mmol/L)	4.8 ± 0.2^b^	4.9 ± 0.3^ab^	4.5 ± 0.2^b^	5.6 ± 0.3^a^	0.032
LDL-C (mmol/L)	2.0 ± 0.3	2.5 ± 0.2	2.6 ± 0.3	2.5 ± 0.2	0.416
VLDL-C (nmol/L)	435.3 ± 24.4^c^	511.9 ± 5.67^b^	423.3 ± 7.1^c^	587.5 ± 10.3^a^	< 0.001

Data are expressed as mean ± SEM. ^a–c^Means within the same row with different superscripts differ. Group C, control group; Group B, added with 4% BSG in the overfeeding stage (days 61–90); Group F, added with 4% BSG in the rearing stage (days 5–60); Group W, added with 4% BSG in the all stage (days 5–90).

### Effect of brewers’ spent grain on the intestinal morphology of Landes geese

The results of the effects of BSG on the morphology of the small intestines of Landes goose were shown in Table 4. In 60 days, no significant difference was observed in VHs and CDs of the duodenum among the four groups of geese (*P* > 0.05). In the jejunum, both VHs and VCRs in groups F and W were significantly increased compared with those of group C (*P* < 0.01). For the ileum, the VCRs of geese fed with BSG were significantly increased in both groups F and W (*P* < 0.01), the VHs in groups F and W were significantly increased compared with group C (*P* < 0.01), while the CDs in groups F and W were significantly lower than that in group C (*P* < 0.05). In 90 days, both VH and VCR of the duodenum in group W were significantly increased compared with group C (*P* < 0.05), while the CD in group W was lower than that in group C (*P* > 0.05). In the jejunum, the VH in group W was significantly higher than those of the other three groups (*P* < 0.05), while no significant difference was observed in the CDs of the four groups of geese (*P* > 0.05). For the ileum, the CD of group F was significantly lower than that in group W (*P* < 0.05), while the VCR was extremely significantly increased (*P* < 0.01).

**TABLE 4:** Effects of brewers’ spent grain (BSG) on the intestinal morphology of the four groups of Landes geese (i.e., groups C, B, F, and W) in 60 and 90 days.

Item		Group C	Group B	Group F	Group W	*P*-value
**Day 60**						
Duodenum (μm)	VH	1083.3 ± 52.1	1091.1 ± 32.9	1145.9 ± 66.8	1140.5 ± 69.9	0.810
	CD	338.3 ± 7.8	332.3 ± 5.4	304.5 ± 8.0	306.5 ± 13.2	0.087
	VCR	3.4 ± 0.1^b^	3.3 ± 0.1^b^	3.9 ± 0.1^a^	3.7 ± 0.1^a^	0.010
Jejunum (μm)	VH	1258.8 ± 38.7^b^	1242.1 ± 50.5^b^	1498.8 ± 39.2^a^	1481.1 ± 53.5^a^	0.003
	CD	297.6 ± 9.7	299.3 ± 14.0	267.5 ± 6.8	262.4 ± 14.1	0.071
	VCR	4.3 ± 0.1^b^	4.2 ± 0.1^b^	5.6 ± 0.1^a^	5.5 ± 0.2^a^	< 0.001
Ileum (μm)	VH	1110.9 ± 49.0	1115.9 ± 29.3	1170.7 ± 36.7	1178.6 ± 48.9	0.476
	CD	267.91 ± 17.7^b^	264.8 ± 12.4^b^	218.4 ± 4.5^b^	221.3 ± 13.9^b^	0.018
	VCR	4.28 ± 0.2^b^	4.2 ± 0.1^b^	5.4 ± 0.1^a^	5.2 ± 0.1^a^	< 0.001
**Day 90**						
Duodenum (μm)	VH	1240.6 ± 137.1^b^	1793.6 ± 24.3^a^	1460.9 ± 66.3^b^	1726.8 ± 212.0^a^	0.014
	CD	223.6 ± 12.1	260.6 ± 17.3	200.2 ± 7.6	213.5 ± 18.7	0.087
	VCR	5.5 ± 0.3^b^	6.9 ± 0.5^ab^	7.3 ± 0.5^ab^	8.2 ± 0.5^a^	0.029
Jejunum (μm)	VH	1579.9 ± 78.0^b^	1544.3 ± 27.7^b^	1495.9 ± 71.1^b^	1772.4 ± 29.0^a^	0.023
	CD	226.9 ± 13.8	266.2 ± 22.5	249.9 ± 24.2	232.5 ± 6.9	0.446
	VCR	7.0 ± 0.7	5.9 ± 0.4	6.1 ± 0.6	7.6 ± 0.1	0.123
Ileum (μm)	VH	1205.7 ± 92.5	1191.9 ± 46.1	1384.9 ± 113.4	1166.4 ± 58.3	0.270
	CD	246.9 ± 11.5^a^	241.1 ± 13.8^a^	196.7 ± 15.9^b^	261.3 ± 26.5^a^	0.015
	VCR	4.9 ± 0.6^b^	5.0 ± 0.1^b^	7.1 ± 0.2^a^	4.8 ± 0.3^b^	0.002

Data are expressed as mean ± SEM. ^a,b^Means within the same row with different superscripts differ. VH, villus height; CD, crypt depth; VCR, villus height/crypt depth ratio. Group C, control group; Group B, added with 4% BSG in the overfeeding stage (days 61–90); Group F, added with 4% BSG in the rearing stage (days 5–60); Group W, added with 4% BSG in the all stage (days 5–90).

### Effect of brewers’ spent grain on the compositions of amino acids in livers of Landes geese

The results of the effects of BSG on the compositions of amino acids in the livers of Landes geese in 90 days were shown in Table 5, revealing no significant difference in the contents of amino acids in the livers of the four groups of Landes geese (*P* > 0.05). It was noted that the contents of six types of amino acids, including glutamic acid, valine, histidine, and the three types of aromatic amino acids (i.e., phenylalanine, tryptophan, and tyrosine) in group W, tended to increase compared with group C.

**TABLE 5:** Effect of brewers’ spent grain (BSG) on the compositions of amino acids in the livers of four groups of Landes geese (i.e., groups C, B, F, and W) in 90 days.

Amino acid (mg/g)	Group C	Group B	Group F	Group W	*P*-value
Aspartic acid	6.3 ± 0.4	6.5 ± 0.2	6.3 ± 0.3	6.5 ± 0.1	0.085
Threonine	3.4 ± 0.1	3.4 ± 0.1	3.4 ± 0.1	3.5 ± 0.1	0.924
Serine	3.2 ± 0.1	3.3 ± 0.1	3.1 ± 0.1	3.3 ± 0.1	0.348
Glutamic acid	10.9 ± 0.1	10.6 ± 0.5	11.9 ± 1.3	11.0 ± 0.7	0.827
Glycine	3.7 ± 0.1	3.6 ± 0.1	3.5 ± 0.1	3.7 ± 0.1	0.385
Alanine	4.6 ± 0.1	4.6 ± 0.2	4.4 ± 0.1	4.6 ± 0.1	0.297
Cystine	1.1 ± 0.2	1.2 ± 0.1	1.3 ± 0.3	1.2 ± 0.0	0.885
Methionine	0.3 ± 0.0	0.4 ± 0.0	0.3 ± 0.0	0.3 ± 0.0	0.969
Isoleucine	3.4 ± 0.1	3.3 ± 0.1	3.5 ± 0.5	3.4 ± 0.1	0.385
Leucine	6.5 ± 0.1	6.5 ± 0.2	6.3 ± 0.2	6.7 ± 0.2	0.268
Tyrosine	2.3 ± 0.0	2.4 ± 0.1	2.2 ± 0.1	2.6 ± 0.1	0.831
Phenylalanine	3.5 ± 0.1	3.5 ± 0.1	3.4 ± 0.1	3.7 ± 0.3	0.349
Lysine	5.3 ± 0.2	5.2 ± 0.3	5.2 ± 0.3	5.4 ± 0.2	0.879
Histidine	1.9 ± 0.1	1.9 ± 0.0	1.8 ± 0.1	2.1 ± 0.1	0.101
Arginine	4.4 ± 0.1	4.3 ± 0.1	4.3 ± 0.2	4.5 ± 0.2	0.606
Proline	3.0 ± 0.1	3.0 ± 0.1	2.9 ± 0.1	3.0 ± 0.0	0.530

Data are expressed as mean ± SEM. Group C, control group; Group B, added with 4% BSG in the overfeeding stage (days 61–90); Group F, added with 4% BSG in the rearing stage (days 5–60); Group W, added with 4% BSG in the all stage (days 5–90).

### Effect of brewers’ spent grain on the compositions of fatty acids in livers of Landes geese

The results of the effects of BSG on the compositions of fatty acids in the livers of Landes geese in 90 days were shown in Table 6. The results revealed the significant decrease in the contents of dodecanoic acid C12:0 (*P* < 0.05) and docosahexaenoic acid C22:2 (*P* < 0.01) in the livers of geese fed with BSG. The content of eleic acid C18:1 was significantly higher in group W than that of group C (*P* < 0.05), while the content of eicosadienoic acid C20:2 was significantly lower in both groups F and W than that of group C (*P* < 0.05). It was noted that compared with group C, the contents of the saturated fatty acid ΣSFA and unsaturated fatty acid ΣSFU in group W tended to decrease and increase, respectively.

**TABLE 6:** Effects of brewers’ spent grain (BSG) on the compositions of fatty acids in the livers of four groups of Landes geese (i.e., groups C, B, F, and W) in 90 days.

Fatty acid	Group C	Group B	Group F	Group W	*P*-value
Decanoic acid C10:0	0.05 ± 0.01	0.06 ± 0.01	0.06 ± 0.02	0.08 ± 0.01	0.293
Eleven carbonic acid C11:0	0.04 ± 0.01	0.04 ± 0.01	0.03 ± 0.01	0.064 ± 0.02	0.185
Dodecanoic acid C12:0	0.06 ± 0.01^a^	0.03 ± 0.00^b^	0.03 ± 0.00^b^	0.04 ± 0.00^b^	0.020
Myristic acid C14:0	0.51 ± 0.01	0.53 ± 0.00	0.42 ± 0.07	0.43 ± 0.05	0.060
Palmitic acid C16:0	22.05 ± 0.35	21.56 ± 0.94	21.41 ± 0.22	20.43 ± 0.65	0.192
Hexadecanoic acid C16:1	2.05 ± 0.71	1.84 ± 0.23	1.65 ± 0.25	1.64 ± 0.06	0.718
Seventeen carbonic acid C17:0	0.07 ± 0.01	0.08 ± 0.03	0.07 ± 0.01	0.08 ± 0.01	0.803
Stearic acid C18:0	14.71 ± 2.21	15.59 ± 1.33	15.31 ± 0.08	15.46 ± 0.11	0.905
Eleic acid C18:1	53.52 ± 1.70^a^	54.09 ± 0.13^a^	56.32 ± 0.47^ab^	57.17 ± 1.03^b^	0.056
Translinoleic acid C18:2	1.58 ± 0.45	1.58 ± 0.14	1.50 ± 0.09	1.61 ± 0.13	0.973
α-linolenic acid C18:3	0.13 ± 0.05	0.09 ± 0.01	0.09 ± 0.01	0.08 ± 0.01	0.371
λ-linolenic acid C18:3	0.09 ± 0.02	0.14 ± 0.02	0.07 ± 0.00	0.08 ± 0.01	0.450
Arachidonic acid C20:0	0.14 ± 0.04	0.17 ± 0.04	0.16 ± 0.01	0.15 ± 0.01	0.766
Eicosadienoic acid C20:2	0.30 ± 0.04^a^	0.31 ± 0.04^a^	0.21 ± 0.02^b^	0.20 ± 0.01^b^	0.047
Eicosatetraenoic acid C20:3	0.19 ± 0.06	0.18 ± 0.11	0.10 ± 0.05	0.12 ± 0.01	0.535
Arachidonic acid C20:4	1.80 ± 0.42	1.64 ± 0.00	1.26 ± 0.12	1.46 ± 0.01	0.115
Eicosapentaenoic acid C20:5	0.08 ± 0.01	0.08 ± 0.06	0.07 ± 0.02	0.07 ± 0.03	0.935
Docosanoic acid C22:0	0.37 ± 0.17	0.19 ± 0.21	0.64 ± 0.00	0.02 ± 0.01	0.104
Docosyl monoenoic acid C22:1	0.04 ± 0.01	0.07 ± 0.01	0.05 ± 0.02	0.06 ± 0.03	0.537
Docosahexadienoic acid C22:2	2.02 ± 0.04^a^	1.61 ± 0.13^b^	1.01 ± 0.04^c^	0.95 ± 0.03^c^	< 0.001
Docosahexaenoic acid C22:6	0.06 ± 0.11	0.08 ± 0.02	0.10 ± 0.01	0.10 ± 0.02	0.114
Tricosanoic acid C23:0	0.13 ± 0.12	0.05 ± 0.03	0.01 ± 0.00	0.14 ± 0.16	0.399
Saturated fatty acids ΣSFA	38.17 ± 2.02	38.30 ± 0.14	37.57 ± 0.18	36.90 ± 0.64	0.638
Unsaturated fatty acid ΣSFU	61.87 ± 1.53	61.70 ± 0.14	62.43 ± 0.18	63.13 ± 0.64	0.423

Data presented as the percentage of the total content of fatty acids. Data are expressed as mean ± SEM. ^a–c^Means within the same row with different superscripts differ Group C, control group; Group B, added with 4% BSG in the overfeeding stage (days 61–90); Group F, added with 4% BSG in the rearing stage (days 5–60); Group W, added with 4% BSG in the all stage (days 5–90).

### Effect of brewers’ spent grain on the bacterial diversity of the intestinal microbiota in Landes geese

**FIGURE 1:**
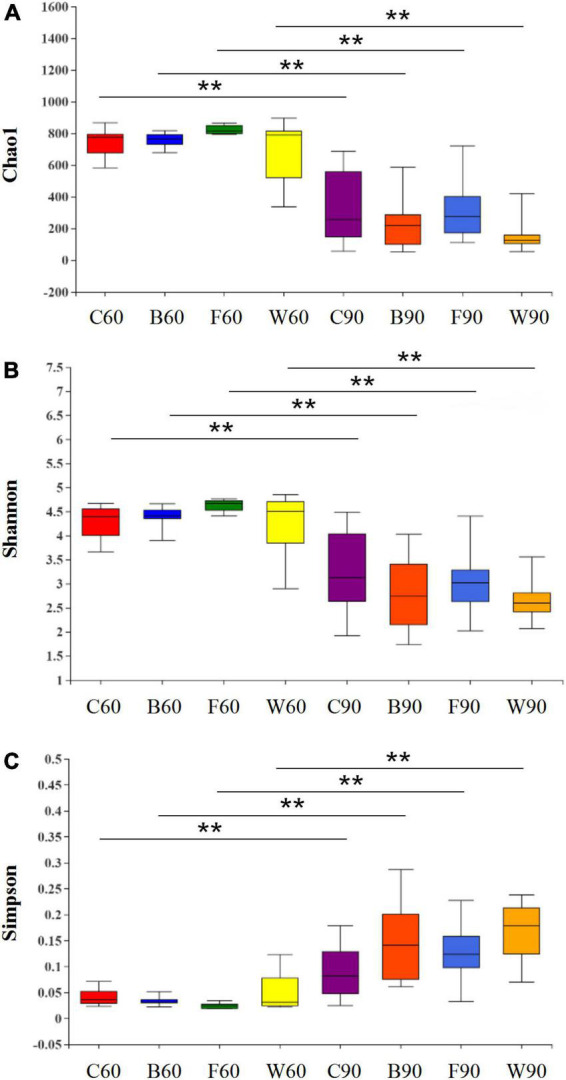
Effect of brewers’ spent grain (BSG) on the alpha diversity of cecal microbiota in Landes geese in 60 and 90 days. **(A)** Chao1 index. **(B)** Shannon index. **(C)** Simpson index. Symbol “**” indicates the significant difference at *P* < 0.01. Group C=, control group; Group B, added with 4% BSG in the overfeeding stage (days 61–90); Group F, added with 4% BSG in the rearing stage (days 5–60); Group W, added with 4% BSG in the all stage (days 5–90).

To explore the effects of BSG on the intestinal microbiota of Landes geese, the 16S rRNA gene was sequenced based on the genomic DNA extracted from the cecal contents of the Landes geese. After screening and splicing of the raw reads, a total of 3,055,821 valid reads were obtained with an average of 50,095 ± 1,155 reads per sample and an average length of 419 ± 0.28 bp (Supplementary Table 5). The clean reads were clustered based on 97% similarity to obtain the representative sequences of OTUs. The rarefaction curve of each sample generally tended to be flat, suggesting that the level of RNA-Seq analysis was sufficient to cover all taxa in the sample (Supplementary Figure 1). Three alpha diversity indices were determined based on the OTUs, i.e., the Chao1 index was evaluated to assess the community richness (Figure 1A), while both the Shannon (Figure 1B) and the Simpson (Figure 1C) indices were measured to evaluate the community diversity. The results showed that in 60 days, no significant difference was observed in the Chao1, Shannon, and Simpson indices in four groups of Landes geese (*P* > 0.05). In 90 days, it was noted that the alpha diversity indices showed an extremely significant difference compared with those of 60 days, showing that the community richness and diversity in cecum of Landes geese were decreased extremely significantly during the overfeeding stage.

**FIGURE 2:**
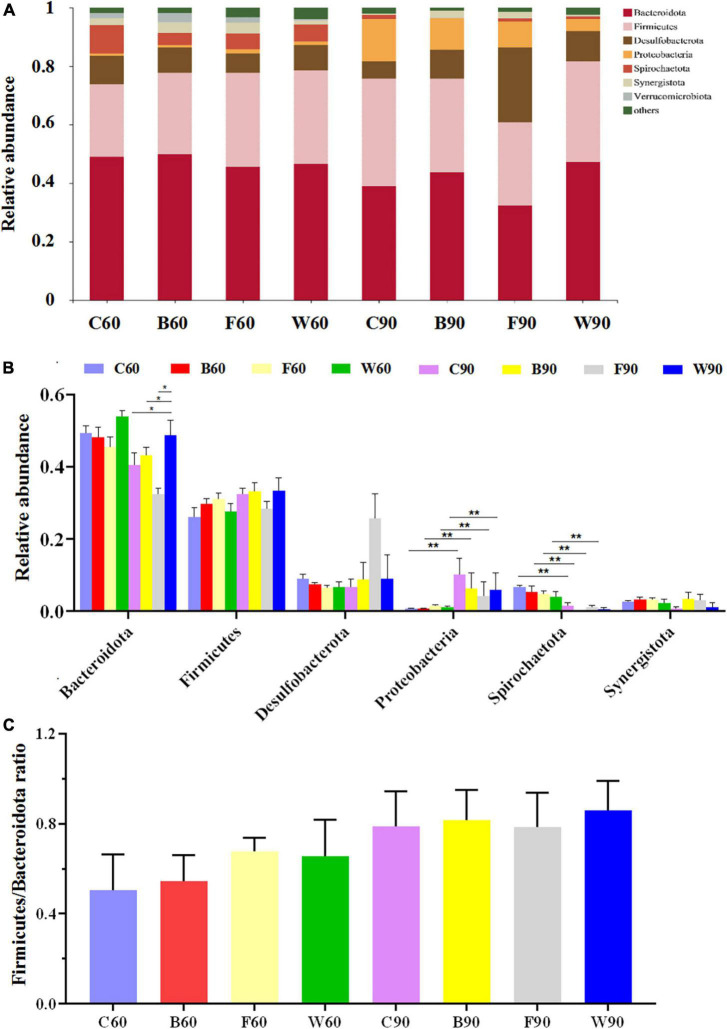
The microbiome compositions in cecum at the phylum level based on the 16S rRNA sequencing in four groups of Landes geese (i.e., groups C, B, F, and W) on both days 60 and 90. **(A)** The relative abundances of the top seven bacterial phyla in the microbiome of Landes geese on days 60 and 90. **(B)** The relative abundances of top seven bacterial phyla among the four groups of Landes geese on days 60 and 90 showing the statistical significance. **(C)** The *Firmicutes/Bacteroides* (F/B) ratio in four groups of Landes geese on days 60 and 90. Values are represented as the mean ± stand error of the mean (SEM) (*n* = 8 geese per group). The significant difference is set at *P* < 0.05 (*) and *P* < 0.01 (**), respectively. Group C, control group; Group B, added with 4% BSG in the overfeeding stage (days 61–90); Group F, added with 4% BSG in the rearing stage (days 5–60); Group W, added with 4% BSG in the all stage (days 5–90).

**FIGURE 3:**
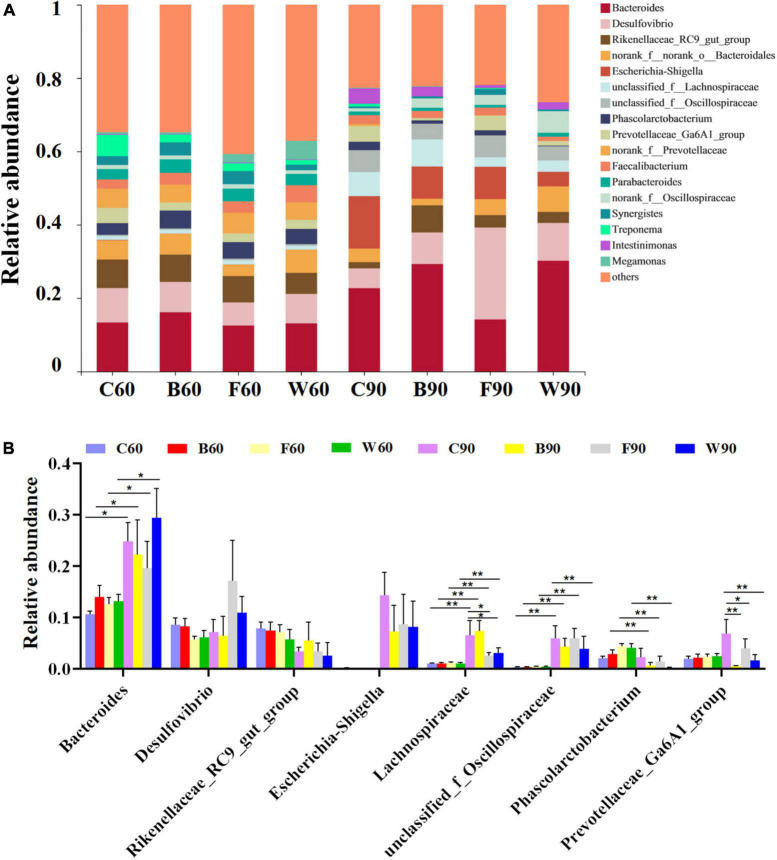
The microbiome compositions in cecum at the genus level based on the 16S rRNA sequencing in four groups of Landes geese (i.e., groups C, B, F, and W) on both day 60 and 90. **(A)** The relative abundances of the top 18 bacterial taxa in the microbiome of the Landes geese on day 60 and 90. **(B)** The relative abundances of the top 18 bacterial taxa in the microbiome of the Landes geese on day 60 and 90 showing the statistical significance. Values are represented as the mean ± stand error of the mean (SEM) (*n* = 8 geese per group). The significant difference is set at *P* < 0.05 (*) and *P* < 0.01 (**), respectively. Group C, control group; Group B, added with 4% BSG in the overfeeding stage (days 61–90); Group F, added with 4% BSG in the rearing stage (days 5–60); Group W, added with 4% BSG in the all stage (days 5–90).

In order to further explore the effects of BSG on the cecal microbiota of Landes geese, the cluster classification of OTUs was investigated at the phylum level (Figures 2A,B). The results revealed *Bacteroides* and *Firmicutes* as the top two bacterial phyla with the highest relative abundances in the cecal microbes of Landes geese during both the rearing and the overfeeding stages. Among the top seven abundant phyla (Figure 2B), the relative abundances of *Proteobacteria* in all four groups of Landes geese were significantly increased in 90 days compared with those in 60 days (*P* < 0.01), whereas the relative abundances of *Spirochaetota* in all four groups of Landes geese were significantly decreased in 90 days compared with those in 60 days (*P* < 0.01). In 90 days, the relative abundance of *Bacteroidetes* in group W was significantly higher than those in groups C, B, and F (*P* < 0.05; Figure 2B). In 60 days, the *Firmicutes/Bacteroidetes* (F/B) ratio in both groups W and F were increased, though not significantly (*P* > 0.05), compared with those of groups C and B (Figure 2C). We then evaluated the variations in the relative abundances of the cecal microbiota in four groups of Landes geese in 60 and 90 days at the genus level (Figures 3A,B). The results showed that the relative abundances of *Bacteroides* (*P* < 0.05), *Lachnospiraceae* (*P* < 0.01), and *unclassified_f_Oscillospiraceae* (*P* < 0.01) in all four groups of Landes geese on day 90 were significantly increased compared to those on day 60. In 60 days, no significant difference was observed in the relative abundance at the genus level of the four groups of Landes geese. In 90 days, the relative abundances of *Escherichia-Shigella* in groups B, F, and W were decreased, though not significantly (*P* > 0.05), compared with that of group C. The relative abundances of *Lachnospiraceae* in groups F and W were significantly lower than that in group C (*P* < 0.05), whereas the relative abundances of *prevotellaceae_Ga6A1_group* in groups B (*P* < 0.01), F (*P* < 0.05), and W (*P* < 0.01) were significantly lower than that in group C.

### Transcriptomics analysis of fatty liver in Landes geese

**FIGURE 4:**
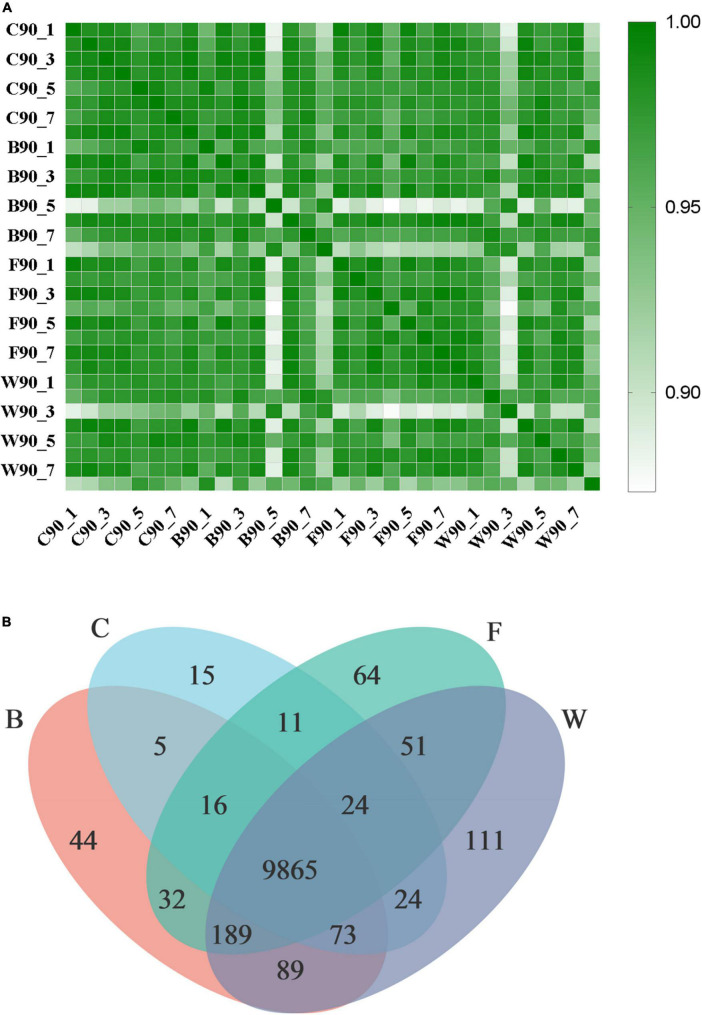
Transcriptomic profiles of liver tissues in four groups of Landes geese (i.e., groups C, B, F, and W) on day 90. **(A)** The Pearson correlation coefficient (*R*^2^) heat map among individual samples in four groups of Landes geese (*P* < 0.01). **(B)** Venn diagram showing the number of expressed genes detected in four groups of Landes geese. Group C, control group; Group B, added with 4% BSG in the overfeeding stage (days 61–90); Group F, added with 4% BSG in the rearing stage (days 5–60); Group W, added with 4% BSG in the all stage (days 5–90).

The transcriptomics analysis was performed based on a total of 32 liver samples (n = 8 samples per group) for four groups of Landes geese (Supplementary Table 6). A total of 225.71 Gb of clean data were obtained with the exclusion of low-quality sequences, while the large portion of the filtered reads (>88%) showed a Phred quality score > 30 (i.e., base Q30). Results showed that the Pearson correlation coefficients (*R*^2^) among the biological repeats in all four groups of Landes geese were high (Figure 4A), suggesting that the RNA-Seq data were reliable for further analyses. The expressions of a total of 10,033, 10,313, 10,252, and 10,426 genes were detected in the liver tissues of four groups of Landes geese, i.e., C, B, F, and W, respectively, with a total of 9,865 genes expressed commonly in all four groups of geese (Figure 4B).

**FIGURE 5:**
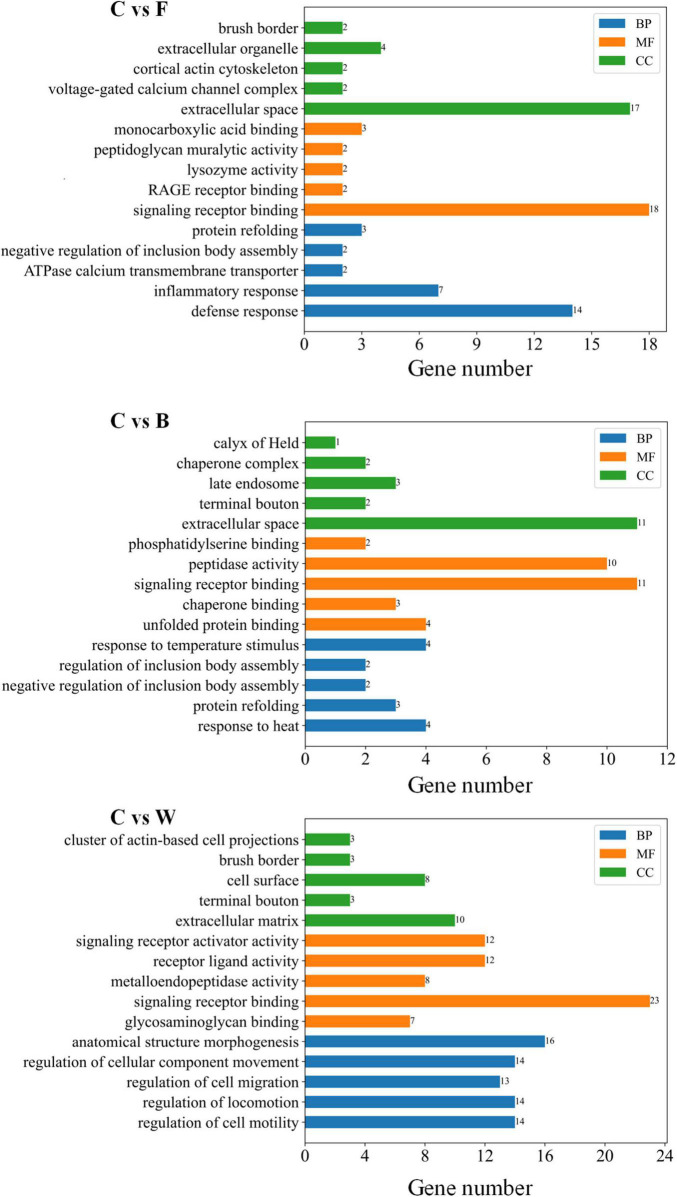
Gene Ontology (GO) annotation of the differentially expressed genes (DEGs) identified between the control (group C) and each of the three experimental groups of Landes geese (i.e., groups F, B, and W) on day 90 showing the top five GO terms in each of the three categories of GO terms, including molecular function (MF), cellular component (CC), and biological process (BP). Group C, control group; Group B, added with 4% BSG in the overfeeding stage (days 61–90); Group F, added with 4% BSG in the rearing stage (days 5–60); Group W, added with 4% BSG in the all stage (days 5–90).

The total number of DEGs varied in different pairwise comparisons (Table 7). A total of 866 DEGs were identified in the four groups of Landes geese using DESeq (Supplementary Table 7). The DEGs identified in the fatty livers of the Landes geese were further annotated based on the GO database (Supplementary Table 8). The results of GO annotation analysis showed that the functions of DEGs were classified into three categories, including biological process (BP), cellular component (CC), and molecular function (MF). Among the top five GO terms in each of these three categories (Figure 5), the highest number of genes were annotated in the GO term of signaling receptor binding in all three pairwise comparisons, followed by the GO terms of extracellular space and anatomical structure morphogenesis.

**TABLE 7:** Differentially expressed genes (DEGs) identified in three pairwise comparisons between the control (group C) and each of the three experiments groups of Landes geese (i.e., groups F, B, and W) treated with brewers’ spent grain (BSG) on day 90 based on transcriptomics analysis.

	Groups C vs. F	Groups C vs. B	Groups C vs. W
Up-regulated	169	143	296
Down-regulated	131	43	84
Total	300	186	380

Group C, control group; Group B, added with 4% BSG in the overfeeding stage (days 61–90); Group F, added with 4% BSG in the rearing stage (days 5–60); Group W, added with 4% BSG in the all stage (days 5–90).

**FIGURE 6:**
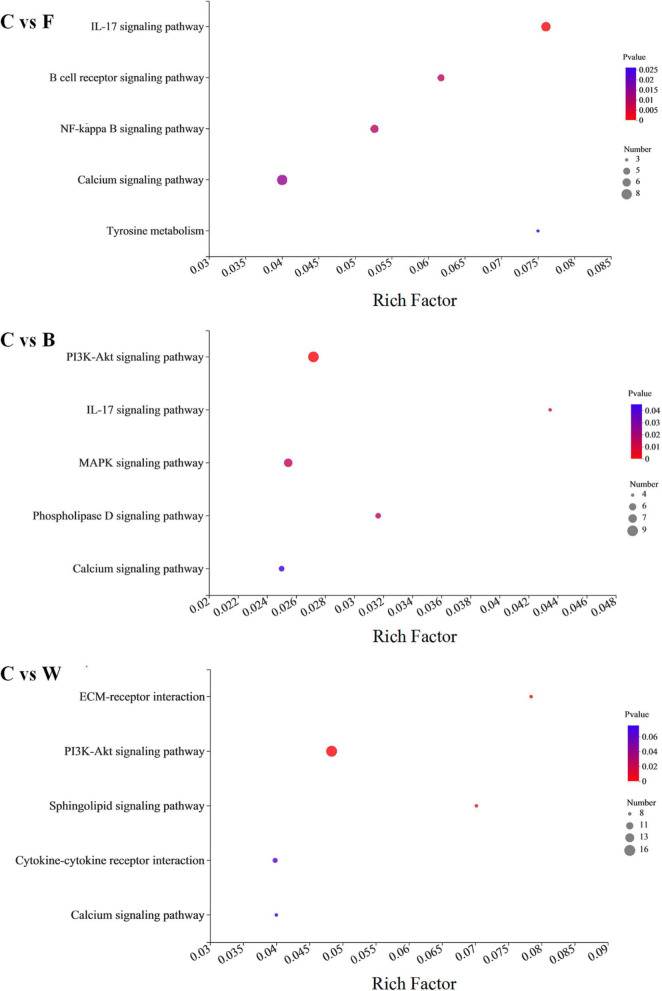
Enrichment analysis based on the Kyoto Encyclopedia of Genes and Genomes (KEGG) database of the differentially expressed genes (DEGs) identified between the control (group C) and each of the three experimental groups of Landes geese (i.e., groups F, B, and W) on day 90. The larger Rich factor value indicates the greater enrichment. The size of the dots indicates proportionally the number of genes enriched in the pathway; the color of the dots corresponds to the *P*-value ranges. Group C, control group; Group B, added with 4% BSG in the overfeeding stage (days 61–90); Group F, added with 4% BSG in the rearing stage (days 5–60); Group W, added with 4% BSG in the all stage (days 5–90).

The enrichment analysis of DEGs identified in the fatty livers of the Landes geese were further performed based on the KEGG database (Figure 6). The results showed that the DEGs identified in group W compared with group C were highly enriched in three metabolic pathways (i.e., ECM-receptor interaction, PI3K-Akt signaling pathway, and sphingolipid signaling pathway), while the other three pathways (i.e., IL-17 signaling pathway, NF-kappa B signaling pathway, and B cell receptor signaling pathway) were highly enriched by DEGs identified in group F compared with group C. The *Q*-values of the KEGG enrichment analysis indicated that most of the genes enriched were involved in the biosynthesis of secondary metabolites.

### Kyoto encyclopedia of genes and genomes enrichment analysis of differentially expressed gene between groups C and W of Landes geese

Due to the extremely significant difference in the liver weight between groups W and C of Landes geese, the KEGG enrichment analysis of the DEGs identified between groups W and C was performed to further evaluate the functions of these genes involved in different metabolic pathways (Table 8). The results showed that in group W, the down-regulated DEGs were significantly enriched in two metabolic pathways (i.e., the protein processing in endoplasmic reticulum and the endocytosis), while the up-regulated DEGs were significantly enriched in four metabolic pathways, including PI3K-Akt signaling pathway, sphingolipid signaling pathway, sphingolipid metabolism, and biosynthesis of unsaturated fatty acids.

**TABLE 8:** Enrichment analysis of differentially expressed genes (DEGs) identified between groups C and W of Landes geese on day 90 based on the Kyoto Encyclopedia of Genes and Genomes (KEGG) database.

DEG	*P*-value	Number of gene	KEGG pathway
Up-regulated		28	
*PPP2R2B, BCL2, PPP2R3B, LAMC2, LAMA2, LAMA4, COL6A1, HGF, THBS2, KIT, FGF10, ANGPT4, IL2RA, COL6A3, PDGFRA*	0.000571	15	PI3K-Akt signaling pathway
*PPP2R2B, PLCB4, BCL2, PPP2R3B, SGPP1, ASAH2, CERS6*	0.003413	7	Sphingolipid signaling pathway
*GAL3ST1, SGPP1, ASAH2, CERS6*	0.011893	4	Sphingolipid metabolism
*ELOVL7, LOC106048282*	0.150318	2	Biosynthesis of unsaturated fatty acids
Down-regulated		8	
*HSPA8, LOC106048968, HSPA2, HSPH1*	0.005821	4	Protein processing in endoplasmic reticulum
*HSPA8, IQSEC3, LOC106044678, HSPA2*	0.021602	4	Endocytosis

Group C, control group; Group W, added with 4% BSG in the all stage (days 5–90).

### Validation of differentially expressed genes

**FIGURE 7:**
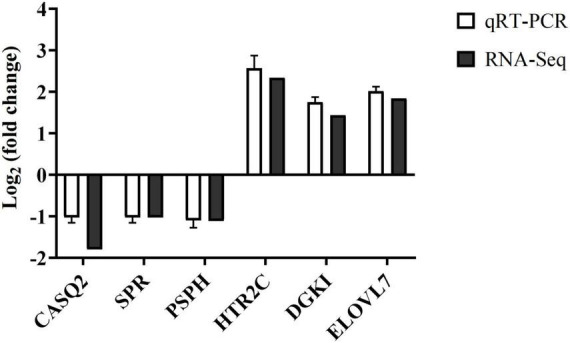
The expression fold changes in six randomly selected differentially expressed genes (DEGs) between groups C and W of Landes geese based on the quantitative real-time PCR (qRT-PCR) analysis validating the expression patterns revealed by the RNA-Seq analysis. Group C, control group; Group W, added with 4% BSG in the all stage (days 5–90).

To validate the identification of DEGs between the groups C and W determined by RNA-Seq, a total of six DEGs (*CASQ2*, *SPR*, *PSPH*, *HTR2C*, *DGKI*, and *ELOVL7*) were randomly selected for qRT-PCR analysis (Figure 7). The results showed that in group W, three genes (i.e., *CASQ2*, *SPR*, and *PSPH*) were down-regulated, while the other three genes (i.e., *HTR2C*, *DGKI*, and *ELOVL7*) were up-regulated. These results were consistent with those of the RNA-Seq analysis, therefore validating the results of the RNA-Seq analysis.

## Discussion

Due to the increasing demand of various types of meat products worldwide, e.g., chicken, beef, and pork, the feed prices have been rapidly escalating. Therefore, it is practically and financially essential to identify feed supplements that are both nutritious and inexpensive. The BSG is the main by-product of the brewing industry and is commonly used in animal feeds, simply because the BSG is not only less expensive than most raw materials available on the market, but also rich in nutrients (Bianco et al., 2020). Furthermore, the BSG may contain microbes capable of producing endogenous ethanol. For example, studies reported a strong correlation between the amount of endogenous ethanol produced in the intestine of obese patients and the pathogenesis of NAFLD (Nair et al., 2001), showing the high relative abundance of alcohol-producing bacteria in the microbiota of NAFLD patients. Moreover, it was suggested that the endogenous ethanol production could promote liver steatosis by stimulating the inflammatory signals (Jandhyala et al., 2015; Boursier and Diehl, 2016). In our study, the beneficial effects of BSG, used as the feed additives, on the liver production performance of Landes geese through the “gut-liver axis” and on the growth performance, intestinal morphological structure, serum biochemical indicators of Landes geese, were investigated based on transcriptomics analysis. Our study provided novel experimental evidence to support the further investigations and applications of BSG in the fat deposition regulation by modulating the gut microbiota in the Landes geese.

### Effects of brewers’ spent grain on the growth performance of Landes geese

Previous studies reported that the addition of 20% brewers’ dried grain to the diets of Vanaraja chicks significantly improved the carcass yield and enhanced profit margins without affecting the growth performance in birds (Denstadli et al., 2010). Furthermore, studies showed that BSG could be used to replace portion of the diet of growing pigs without causing adverse effects on daily weight gain and economic benefits (Mukasafari et al., 2018). However, these results were not consistent with the findings revealed in our study. In particular, our results showed that the addition of BSG in the feeds caused an increasing trend in both BW and ADG of Landes geese during the rearing period (Table 2). These conflicting results were probably because that the BSG used in our study was already fermented by microorganisms, and there were a large number of probiotics and enzymes in the fermented BSG, which could improve the digestibility of nutritional components, ultimately reducing the anti-nutritional factors and harmful components in the raw materials, as previously reported (Al-Khalaifah et al., 2020). Moreover, studies showed that the BSG could produce a type of unique fragrance derived from the probiotic fermentation, improving the feed intake and weight gain of lambs (Frasson et al., 2018), while the addition of 40% fermented brewers’ dried grain to the diet of Meihua pigs resulted in rapid weight gain and high feed remuneration, largely decreasing the feeding costs of Meihua pigs as well as effectively preventing diarrhea and reducing stool odor (Wu et al., 2018). These results suggested that the fermented BSG not only reduced the feeding costs but also improved the animal growth performance, showing significant potential of feed additives in animal feed industry.

### Effects of brewers’ spent grain on the fatty liver deposition in Landes geese

It is well-known that beer fermentation is a metabolic process converting both monosaccharides and disaccharides into ethanol under anaerobic or micro-oxygen conditions by microorganisms such as yeast, while a large number of alcohol-producing microorganisms are identified in the BSG (Zabed et al., 2017; Rojas-Chamorro et al., 2020). For example, studies showed that the high gravity brewing yeast *Saccharomyces cerevisiae* BLGII 1762 and *S. cerevisiae* PE-2 isolated from the bioethanol industry produced ethanol with yields of 42.27 g/L and 40.3 g/L, respectively (Pinheiro et al., 2019). Furthermore, it was previously reported that the ethanol produced by high alcohol-producing bacteria was an important factor causing liver lipid accumulation and ultimately NAFLD (Chen et al., 2020). Studies have shown that in the obese mice, ethanol could be detected in exhaled gas even in the absence of ethanol intake, and the increased level of exhaled ethanol indicated the increased production of ethanol by the gut microbiota, which could contribute to the development of fatty liver (Cope et al., 2000). Moreover, studies showed that gut microbiota rich in alcohol-producing bacteria (e.g., *Klebsiella pneumoniae*) constantly produced more alcohol through 2,3-butanediol fermentation pathway involved in fatty liver disease (Li N. N. et al., 2021). In our study, the results showed that the addition of BSG in feeds caused significant increase in liver weight of Landes geese during the overfeeding stage (Table 2). This could be explained by the presence of endogenous ethanol-producing microorganisms in BSG causing the increased liver weight, suggesting the positive effect of microorganisms capable of producing endogenous ethanol to enhance the fatty liver deposition in Landes geese.

### Effects of brewers’ spent grain on the serum biochemical index in Landes geese

Blood biochemical indices are generally considered sensitive indicators used to evaluate the body’s systemic or local metabolic changes and the physiological functions of various types of tissues (Kani et al., 2013). In our study, the BSG significantly reduced the serum GLU level during the rearing stage (Table 3), while previous studies identified the correlation between the increased level of hunger and the observed decrease in blood GLU level (Campfield and Smith, 2003). Therefore, the significantly higher ADG in geese fed with BSG was not only due to the fermentation process that improved the palatability of the feeds, but also associated with the lower blood GLU level. Both AST and ALT are two important amino acid transferases that are generally detected at low levels in the blood, released from cells into the blood when tissue damage or necrosis occurs in organs such as the liver, resulting in the increased enzymatic activities in the serum (Kunde et al., 2005). Therefore, the serum levels of AST and ALT could reflect the health status of the liver and heart. In our study, the increased serum levels of both ALT and AST were observed in 90-day-old Landes geese compared with those of the 60-day-old geese (Table 3), suggesting that the force-feeding process caused the abnormal liver function, i.e., formation of fatty liver, as reported previously (Locsmándi et al., 2007).

The lipids synthesized in goose liver are either stored in hepatocytes as cytoplasmic droplets or secreted as lipoproteins into blood (Hermier, 1997). Our results showed that the overfeeding of Landes geese with BSG caused the significantly increased serum lipid concentration in Landes geese, with the contents of both HDL and VLDL increased considerably. However, no significant difference was observed in the contents of TG and CHO among the four groups of Landes geese (Table 3). These results were consistent with those reported previously. For example, due to the dramatic increase in new hepatic lipogenesis caused by overfeeding, both TG and CHO did not fully enter the secretory pathway with a large amount of TG remained and stored in the liver (Liu et al., 2020). Studies have shown that HDL could lower the blood cholesterol level mainly by transporting cholesterol from peripheral tissues and plasma to the liver for both metabolism and excretion through apolipoproteins (i.e., APOA1 and APOC3), while as the main form of endogenous cholesterol synthesized by the liver and transported to other tissues, the LDL is rich in cholesterol esters to release cholesterol through its APOB100 protein binding to receptors on non-hepatocyte plasma membranes (Ikonen, 2008; Min et al., 2012). Furthermore, studies reported that the increased rate of cholesterol synthesis in the liver in comparison to that of VLDL-C secretion resulted in a large amount of cholesterol deposition in the liver (Han et al., 2008; Griffin et al., 2009). Moreover, a positive correlation between VLDL-C concentration and liver weight was revealed in Polish geese (Mourot et al., 2000). These results indicated that the high VLDL-C concentration indicated the enhanced fat deposition in the liver tissue. In our study, increased HDL-C and VLDL-C secretion rates caused by the addition of BSG strongly indicated that BSG could make significant impact on the lipoproteins, which in turn affected the deposition of TG in the liver, ultimately increasing the weight of the fatty liver in Landes geese.

### Effects of brewers’ spent grain on the intestinal morphology in Landes geese

The small intestine is the main organ for digestion and absorption, which are crucial to the growth performance of the Landes geese. Studies have shown that as the main microstructures functioning in nutrient absorption and transport, the villi with increased height obtained an enhanced nutrient absorption capacity, while the crypt depth (CD) reflected the turnover rate of the intestinal epithelium (Gu et al., 2020). Therefore, the villus height/crypt depth ratio (VCR) is commonly used as an important factor for evaluating the absorption capacity of small intestine. For example, it was reported that the addition of wine lees and soluble matter to broiler diets significantly (*P* < 0.05) increased the jejunal villus height (VH) (Alizadeh et al., 2016), while the supplementation of diets with 2.5% fermented feed increased the proximal jejunal VH in geese, ultimately causing the beneficial effect on growth performance and nutrient digestibility (Yan et al., 2019). These results were consistent with our findings, showing that the BSG treatment markedly increased the VCRs of the duodenum, jejunum, and ileum of Landes geese (Table 4). These results strongly indicated that the fermented BSG enhanced the villus development and the absorption of nutrients in the small intestine, leading to the significantly increased BW and ADG in the Landes geese fed with BSG in comparison with those of the control group.

### Effects of brewers’ spent grain on the compositions of liver amino acids and fatty acids in Landes geese

As two of the important indicators for evaluating the nutritional value of the goose liver, both the compositions and ratios of various amino acids and fatty acids affect the physicochemical properties and flavor of goose liver (Liu et al., 2011; Zhu et al., 2011). Amino acids could activate G protein-coupled receptors (GPCRs) to control the ion channels in the umami and sweet taste pathways, directly affecting the mammalian taste ability (Oike et al., 2006). In our study, no significant differences were observed in each amino acid in the livers of four groups of Landes geese (Table 5). However, the levels of glutamic acid, valine, histidine, and three types of aromatic amino acids (i.e., phenylalanine, tryptophan, and tyrosine) in the experimental groups of geese were slightly increased compared with those of the control group, suggesting that the addition of BSG could improve the flavor of goose liver.

As people’s living standards rapidly improve, the polyunsaturated fatty acids (PUFAs) are increasingly demanded in the world market due to their health benefits. As the essential types of nutrient, the PUFAs have shown a preventive effect against chronic diseases (Gillingham et al., 2011). Therefore, it is now widely recommended to increase the intake of PUFAs in human diets (Chen et al., 2016). For example, it was reported that the addition of distillers’ dried grains in the laying duck diet significantly (*P* < 0.01) increased the ratio of oleic acid (C18:1) and the total monounsaturated fatty acids in egg yolk (Ruan et al., 2018), while the concentration of the total PUFAs in the bull muscles fed with distillers’ grains was higher than that of the control group (He et al., 2015). In our study, the results showed that both C16:0 and C18:1 were the predominant fatty acids in the liver of Landes geese, with C18:1 accounted for more than 50% of the total fatty acids, while the addition of BSG increased the content of C18:1 to the highest levels in goose livers of group W compared to the control group (Table 6). In summary, the addition of BSG to the diets of Landes geese significantly increased the concentrations of unsaturated fatty acids in the livers of Landes geese.

### Effects of brewers’ spent grain on the microbial diversity of the intestinal microbiota in Landes geese

It is important to understand the relationship between gut microbiota and growth performance in order to effectively improve the growth and production performance of Landes geese. In our study, the bacterial communities of the cecal samples were comparatively investigated among the four groups of geese on both day 60 and 90. The results showed that species richness and diversity of the microbial communities were not significantly altered by the addition of BSG during the entire experiment of both the rearing and overfeeding stages (Figure 1). However, the alpha diversity indices (i.e., Chao1, Shannon, and Simpson) of Landes geese were decreased after the overfeeding stage. Studies reported that in both the ileal and cecal samples, the probiotic addition showed no significant effect on species richness and diversity before overfeeding, whereas both diversity and species richness tended to decrease after the overfeeding (Even et al., 2018). These results were consistent with the findings revealed in our study, suggesting that the overfeeding could modulate the intestinal microbiota in Landes geese.

Our results revealed that all cecal samples were relatively dominated by both Bacteroidota and Firmicutes at the phylum level (Figure 2A), similar to the results previously reported (Waite and Taylor, 2014), and were dominated by *Bacteroides*, *Desulfovibrio*, and *Rikenellaceae_RC9_gut_group* at the genus level (Figure 3A). As a generally predominant genus in the poultry intestine, *Bacteroides* shows its unique physiological characteristics (Aruwa et al., 2021), e.g., regulating the intestinal redox level (Wexler and Goodman, 2017), participating in the carbohydrate metabolism, and generating the main end-products (i.e., acetate, propionate, and butyrate) in sugar fermentation (Fu et al., 2019). Studies have shown that the relative abundance of *Bacteroidetes* was increased in the laying hens as a model group for NASH (Hamid et al., 2019). Furthermore, it was reported that the gut *Firmicutes*/*Bacteroidetes* ratio was positively correlated with steatosis in the obese patient group (Jasirwan et al., 2021). Moreover, studies have shown that an increase in the proportion of *Proteobacteria* is the most significant change in gut-liver axis induced hepatic steatosis in mice (Vasques-Monteiro et al., 2021). In our study, the relative abundances of *Proteobacteria* in all four groups of Landes geese were significantly increased after the overfeeding stage (Figure 2B). However, there was no significant difference in the relative abundance of *Proteobacteria* between the four groups of Landers geese (i.e., groups C, B, F, and W) in 90 days. These results suggested that future studies should also evaluate the microbial taxa at both genus and family levels in the phylum *Proteobacteria* in order to explore the development of NAFLD. Studies have shown that *Phascolarctobacterium* was a substantially main acetate propionate producer that could be dramatically increased by berberine and metformin (Wu et al., 2017). Moreover, it was suggested that the high relative abundance of *Phascolarctobacterium* in low-aerobic-capacity rats could contribute to their susceptibility to acute high fat diet-induced hepatic steatosis (Panasevich et al., 2016). Furthermore, *Lachnospiraceae* have been recognized as fermentative commensals that produce short-chain fatty acids (SCFAs), which are involved in the maintenance of intestinal health (Sorbara et al., 2020). Our results showed that the relative abundances of both *Phascolarctobacterium* and *Lachnospiraceae* were increased after the overfeeding stage (Figure 3B). However, the relative abundances of *Phascolarctobacterium* and *Lachnospiraceae* were decreased with the addition of BSG in feeds, which was inconsistent with our results of growth performance in Landes geese, probably due to the consumption of the nutrients by *Bacillus subtilis* and yeast entering the intestinal tract, suggesting that feeding fermented BSG was not conducive to the growth of *Phascolarctobacterium* and *Lachnospiraceae*. Indeed, studies showed that the relative abundance of *Phascolarctobacterium* and *Lachnospiraceae_uncultured* were decreased by adding fermented feed to geese diets (Yuan et al., 2019). Furthermore, studies showed that both β-glucans and arabinoxylan inhibited the proliferation of *Lachnospiraceae_XPB_1014_group* to enhance the production of butyrate (Bai et al., 2021), while the BSG contained large amounts of β-glucans and arabinoxylan.

It has been reported that the high-meat protein diet could increase the relative abundances of *Desulfovibrio* in both cecum and colon to cause the metabolic defects in liver (Shi et al., 2020), while a high relative abundance of *Desulfovibrio* was revealed in the pig model of NASH (Panasevich et al., 2018). Furthermore, the richness of *Desulfovibrio*_*Otu047* was increased with the increased activities of the NAFLD-HCC process (Zhang et al., 2021a). The Shiga toxin-producing *Escherichia–Shigella* are pathogenic bacteria that cause the bloody diarrheal diseases of bacillary dysentery and hemorrhagic colitis (Lee et al., 2020). It was reported that the harmful bacteria such as *Escherichia–Shigella* and *Helicobacter* were prevalent in the intestine of rats with alcohol-related liver injury (Yu et al., 2020), while the *Prevotellaceae_Ga6A1_group* was enriched in the gastric mucosal microbiota of patients with gastric intraepithelial neoplasia (Zhang et al., 2021b). In our study, no significant differences were observed in the relative abundances of these three groups of bacteria (i.e., *Desulfovibrio, Escherichia–Shigella*, and *Prevotellaceae_Ga6A1_group*) among the four groups of Landes geese during the rearing stage, whereas the relative abundances of these taxa were significantly increased during the overfeeding stage as the fatty liver was formed (Figure 3B). The addition of BSG caused the decrease in the relative abundances of these three groups of bacteria, suggesting that the probiotic properties of fermented BSG were involved in the protective mechanism in Landes geese preventing the progression of steatosis to steatohepatitis in their livers.

### Regulatory functions of brewers’ spent grain in the fatty liver development in Landes geese based on the transcriptomics analysis

It has been reported that the fatty livers of geese composed of adipose tissue are achieved with significant weight gain after overfeeding (Wang et al., 2019). This phenomenon is generally considered being related to the long-distance migration of migratory birds. Domestic geese are descendants of migratory birds, suggesting their high tolerance for energy intake (Lu et al., 2015). Our study indicated that the addition of BSG in feed caused an increasing trend in liver weight, suggesting the intrinsic variations in transcriptional regulation during the liver development. Furthermore, our results of KEGG enrichment analysis showed that with the addition of BSG only in the rearing stage (i.e., group F), the DEGs were most significantly enriched in three metabolic pathways, including the NF-kappa B signaling pathway, IL-17 signaling pathway, and B cell receptor signaling pathway (Figure 6). Previous studies showed that the transcription factor NF-κB played a key role in the host response to microbial infection by coordinating innate and adaptive immune functions (Peng et al., 2020), while the IL-17 was a host defense cytokine located in barrier mucosal tissues, playing an important role in immunity against fungal and other extracellular pathogens (Conti et al., 2009). These results suggested that the feeding of the Landes geese with BSG at the rearing stage generated a positive regulatory effect on activating the immune system in the geese. When the BSG was added only in the overfeeding stage (i.e., group B), the DEGs were most significantly enriched in the PI3K-Akt signaling pathway, MAPK signaling pathway, and phospholipase D signaling pathway, while the Landes geese were fed with BSG in both the rearing and overfeeding stages (i.e., group W), the DEGs were most significantly enriched in PI3K-Akt signaling pathway, sphingolipid signaling pathway, and cholinergic synapse (Figure 6). Previous studies showed that the formation of fatty liver in geese by overfeeding was accompanied by the activation of the PI3K-Akt-MTOR pathway, suggesting that the PI3K-Akt-MTOR pathway played a key role in regulating the lipid metabolism (Han et al., 2015). Furthermore, it was reported that with the essential feature of an aliphatic amino alcohol sphingolipid skeleton, the sphingolipids were involved in the development of NAFLD (Gorden et al., 2015). Moreover, the sphingolipids were reported to play the critical roles in the physiological functions of *Bacteroidetes*, which were capable of performing activities related to symbiotic functions in the gut (An et al., 2011). In our study, the results showed that the sphingolipid signaling pathway was significantly enriched in the livers of Landes geese of group W, suggesting that the addition of BSG aggravated the hepatic steatosis, which was probably caused by the intestinal bacteria. Notably, our results revealed a relatively high abundance of bacteria in group W of Landes geese in comparison with group C. These results were consistent with those derived from the liver transcriptomics analysis in our study.

Our results of differential gene expression analysis showed that the expressions of several genes, e.g., *BCL-2*, *ELOVL7*, *FGF10*, and *HGF*, were up-regulated in liver tissues when BSG was added to feed during both the rearing and overfeeding stages (i.e., group W). It was reported that the activation of the BCL-2 protein family induced the hepatocyte apoptosis, which played important roles in the formation of NAFLD (Kanda et al., 2018). Compared with the NAFLD patients, the NASH patients showed lowered level of the anti-apoptotic BCL-2 protein, with a strong negative correlation between BCL-2 level and lobular inflammation (El Bassat et al., 2014), which could be explained by the increased BCL-2 concentration in hepatic steatosis, while the hepatic steatosis was a detoxification process because the free fatty acids (FFA) were directly cytotoxic to hepatocytes. The anti-apoptotic processes enhanced the triglyceride formation and inhibited the FFA toxicity, while the high levels of anti-apoptotic BCL-2 revealed in NAFLD suggested its protective role in disease progression (Tarantino et al., 2011). These results indicated that in our study, the significantly up-regulated expression of *BCL-2* in the livers of Landes geese in group W compared to group C suggested that the BSG supplementation reduced the liver damage (i.e., liver inflammation pressure) and enhanced deposition of lipids in the livers. The FA elongase, also known as a long-chain fatty acid-like fatty acid elongase (ELOVL), cooperates with desaturases to synthesize either monounsaturated fatty acids or PUFAs. Studies have shown that the FA elongase 7 (ELOVL7) played an important role in the synthesis of long-chain saturated fatty acids (Green et al., 2010; Naganuma et al., 2011), while the overexpression of *ELOVL7* significantly decreased the concentrations of palmitoleic acid (C16:1) and increased the concentrations of vaccine (C18:1) (Shi et al., 2019). These results were consistent with the findings revealed in our study (Table 6).

The fibroblast growth factors (FGFs) are a polypeptide family, with the FGF10 as an important intercellular signaling molecule in adipogenesis and highly expressed in adipose tissue (Itoh and Ornitz, 2011). Furthermore, the FGF10 stimulated cell proliferation of white adipose tissue and played an essential role in adipogenesis (Matsubara et al., 2013). It was reported that the overexpression of *FGF10* mainly activated the PI3K-Akt pathway to play a protective role in mouse liver (Li S. T. et al., 2021). These results suggested that the hepatocyte growth factor (HGF) played an important role in liver adipose tissue. In our study, the significantly up-regulated expressions of the *FGF10* and *HGF* genes in the group W of Landes geese indicated the enhanced synthesis of active glycogen and protein and lipid differentiation in the livers of these geese. Our results were consistent with those previously reported, strongly indicating that FGF10 was involved in adipose expression and played an important role in the formation of fatty liver in geese.

The heat shock proteins (HSPs) constitute a large family of highly homologous chaperone proteins that are induced in response to elevated temperature, and more generally in response to environmental, physical, and chemical stresses (Bonam et al., 2019). Previous studies showed that the heat stress could increase the HSP70 levels in goats, while the cell metabolism was accelerated and the respiratory function was enhanced (Dangi et al., 2015). Furthermore, it was reported that the probiotics reduced the *HSP60* gene and protein expression levels in mouse model of alcoholic liver (Barone et al., 2016). Our results of the KEGG enrichment analysis showed that under the treatment of BSG, the down-regulated genes were predominantly enriched in the protein processing in endoplasmic reticulum and endocytosis (Table 8), in particular, the expressions of *HSPA8*, *HSPA2*, and *HSPH1* were significantly down-regulated in the group W of Landes geese. These results indicated that overfeeding could lead to chronic stress in the Landes geese. However, the heat stress genes were down-regulated in the group W of Landes geese, probably due to the alleviation of oxidative stress caused by the microorganisms in the fermented BSG.

In recent years, due to the rapid increases in global feed prices, there has been a rapidly growing interest in the use of industrial by-products as alternative feeds. However, the by-products of grains are generally not favored due to their rich anti-nutritional factors and the difficulties to preserve under regular practical conditions (Jackowski et al., 2020). With the rapid development of the microbial fermentation technology, the solutions to the problems of waste of resources and the production performance of geese have been gradually identified and established. In our study, the results showed that the addition of BSG in feed of Landes geese generated the optimal effect on liver fat deposition in the group W of Landes geese during the entire experiment, probably due to the production of endogenous ethanol by the microorganisms in BSG and the secondary sphingolipid metabolism involved in the adaptation of the gut to overfeeding and maintaining the structural integrity of the gut, as reported previously (Gu et al., 2020). Consistently, the cecal microbiota was closely involved in the sphingolipid metabolism (Li et al., 2019). These results suggested that the cecal microbiota contributed significantly to the regulation of fat deposition, benefiting the development of technical strategies for the significant advancement of the foie gras industry.

## Conclusion

In conclusion, our study revealed the beneficial effects of BSG as the feed additives on the growth performance during the rearing stage (days 5–60) of Landes geese, with both the BW and ADG of the Landes geese significantly increased, whereas the optimal effect of BSG on liver fat deposition was achieved during the overfeeding stage (days 61–90). The intestinal microbiota compositions of Landes geese on day 90 were altered by the addition of BSG, mainly increasing the relative abundance of *Bacteroides* and inhibiting Gram-negative pathogenic bacteria such as members of *prevotellaceae_Ga6A1_group.* Results of the transcriptomics analysis showed that addition of BSG to Landes geese diets altered the expression of genes involved in PI3K-Akt signaling pathway and sphingolipid metabolism in the liver. These beneficial effects were probably caused by either the endogenous ethanol-producing microorganisms in BSG or the fermentation products of BSG mitigating the development of NAFLD. Further studies are necessary to explicitly explore the molecular mechanism underlying the formation of NAFLD with the involvement of microorganisms in BSG. Our study provided novel experimental evidence based on the cecal microbiota to support the investigations and applications of BSG in the regulation of the fat deposition by modulating the gut microbiota in the Landes geese.

## Data availability statement

The datasets presented in this study can be found in online repositories. The names of the repository/repositories and accession number(s) can be found in the article/Supplementary material.

## Ethics statement

This animal study was reviewed and approved by The Committee on the Ethics of Animal Experiments of the College of Veterinary Medicine, Huazhong Agricultural University (NO. HZAUGE–2020–0001).

## Author contributions

PX, DS, ZZ, ZL, and YX: conceptualization. PX, YH, PC, XW, SL, JW, FM, and YX: methodology. PX and PC: validation. PX: formal analysis, investigation, and data curation. YX: resources. PX and YH: software and writing—original draft preparation. YX and SC: writing—review and editing. All authors have read and agreed to the published version of the manuscript.

## Footnotes

1
http://www.drive5.com/uparse/

2
http://rdp.cme.msu.edu/

3
http://bioconductor.org/packages/stats/bioc/DESeq2/

4
http://deweylab.biostat.wisc.edu/rsem/

5
http://geneontology.org/

6
https://www.genome.jp/kegg/

7
https://github.com/tanghaibao/goatools

8
http://kobas.cbi.pku.edu.cn

## Supplementary material

Supplementary Figure 1 The rarefaction curves of the cecal microbial communities in the control (group C) and three experimental groups (B, F, and W) of Landes geese in 60 and 90 days. Group B, added with 4% BSG in the overfeeding stage (days 61–90); Group F, added with 4% BSG in the rearing stage (days 5–60); Group W, added with 4% BSG in the all stage (days 5–90).

Supplementary Table 1 Ingredients and nutrient compositions of diets (%) in the control (group C) and three experimental groups (B, F, and W) of Landes geese during the rearing stage (days 5–15).

Supplementary Table 2 Ingredients and nutrient compositions of diets (%) in the control (group C) and three experimental groups (B, F, and W) of Landes geese during the rearing stage (days 16–60).

Supplementary Table 3 Ingredients and nutrient compositions of diets (%) in the control (group C) and three experimental groups (B, F, and W) of Landes geese during the overfeeding stage (days 61–90).

Supplementary Table 4 Primers and their sequences used in the quantitative real-time PCR (qRT-PCR) analysis in this study. “F” and “R” at the end of each primer name indicate the forward and reverse primers, respectively.

Supplementary Table 5 Summary of 16S rRNA sequencing data based on the cecal contents in the control (group C) and three experimental groups (B, F, and W) of Landes geese in 60 and 90 days.

Supplementary Table 6 Summary of sequencing data based on the liver transcriptomics analysis the control (group C) and three experimental groups (B, F, and W) of Landes geese in 60 and 90 days.

Supplementary Table 7 Differentially expressed genes between the control and experimental groups.

Supplementary Table 8 Gene Ontology terms annotated based on the DEGs between control and experimental groups.

## Abbreviations

BSG: brewers’ spent grain
ALT: alanine aminotransferase
AST: aspartate aminotransferase
AKP: alkaline phosphatase
ACP: acid phosphatase
GLU: glucose
TG: triglycerides
CHO: cholesterol
HDL-C: high density liptein cholesterol
LDL-C: low density liptein cholesterol
VLDL: very low-density lipoprotein
VH: villus height
CD: crypt depth
VCR: villus height/crypt depth
BW: body weight
LW: liver weight
ADG: average daily gain
TPM: transcripts per million
DEG: differentially expressed gene
GO: Gene Ontology
KEGG: Kyoto Encyclopedia of Genes and Genomes
SCFA: short-chain fatty acids
NAFLD: non-alcoholic fatty liver disease
FGFs: fibroblast growth factors
HSP: heat shock proteins
ELOVL7: fatty acid elongase 7.
