# Supplementary material for: Regulation of the cecal microbiota community and the fatty liver deposition by the addition of brewers’ spent grain to feed of Landes geese
Source: Front Microbiol. 2022 Sep 20;13:970563. doi: 10.3389/fmicb.2022.970563 (PMC9530188; doi:10.3389/fmicb.2022.970563)
Supplement: Supplementary Figure 1 — The rarefaction curves of the cecal microbial communities in the control (group C) and three experimental groups (B, F, and W) of Landes geese in 60 and 90 days. Group B, added with 4% BSG in the overfeeding stage (days 61–90); Group F, added with 4% BSG in the rearing stage (days 5–60); Group W, added with 4% BSG in the all stage (days 5–90). [file Data_Sheet_1.ZIP › Supplementary Material/Figure S1.doc]

**Supplementary Figure 1.** The rarefaction curves of the cecal microbial communities in the control (group C) and three experimental groups (B, F, and W) of Landes geese in 60 and 90 days
