# Supplementary material for: Regulation of the cecal microbiota community and the fatty liver deposition by the addition of brewers’ spent grain to feed of Landes geese
Source: Front Microbiol. 2022 Sep 20;13:970563. doi: 10.3389/fmicb.2022.970563 (PMC9530188; doi:10.3389/fmicb.2022.970563)
Supplement: Supplementary Figure 1 — The rarefaction curves of the cecal microbial communities in the control (group C) and three experimental groups (B, F, and W) of Landes geese in 60 and 90 days. Group B, added with 4% BSG in the overfeeding stage (days 61–90); Group F, added with 4% BSG in the rearing stage (days 5–60); Group W, added with 4% BSG in the all stage (days 5–90). [file Data_Sheet_1.ZIP › Supplementary Material/Tables S1-S6 .doc]

**Supplementary Table 1. Ingredients and nutrient compositions of diets (%) in the control (group C) and three experimental groups (B, F, and W) of Landes geese during the rearing stage (day 5 to 15).**

| **Ingredients** | **Group C** | **Group B** | **Group F** | **Group W** |
| --- | --- | --- | --- | --- |
| DDGC | 15.00 | 15.00 | 15.00 | 15.00 |
| Wheat | 12.31 | 12.31 | 12.31 | 12.31 |
| Soybean meal | 9.95 | 9.95 | 9.95 | 9.95 |
| Rice bran | 7.50 | 7.50 | 7.50 | 7.50 |
| Paddy | 8.00 | 8.00 | 8.00 | 8.00 |
| Corn | 5.00 | 5.00 | 5.00 | 5.00 |
| Brown rice mixture | 35.00 | 35.00 | 35.00 | 35.00 |
| Meat and bone powder | 2.50 | 2.50 | 2.50 | 2.50 |
| Fine stone powder | 0.51 | 0.51 | 0.51 | 0.51 |
| CaHPO4 | 0.62 | 0.62 | 0.62 | 0.62 |
| BSG fermented | 0.00 | 0.00 | 4.00 | 4.00 |
| Lard | 0.50 | 0.50 | 0.50 | 0.50 |
| Methionine | 0.24 | 0.24 | 0.24 | 0.24 |
| Lysine | 0.79 | 0.79 | 0.79 | 0.79 |
| Threonine | 0.15 | 0.15 | 0.15 | 0.15 |
| Enzymatic Peptide | 1.00 | 1.00 | 1.00 | 1.00 |
| NaCl | 0.20 | 0.20 | 0.20 | 0.20 |
| Choline chloride | 0.10 | 0.10 | 0.10 | 0.10 |
| NaHCO_3_ | 0.20 | 0.20 | 0.20 | 0.20 |
| Enzyme complex | 0.03 | 0.03 | 0.03 | 0.03 |
| Premix ^1^ | 0.40 | 0.40 | 0.40 | 0.40 |
| **Total** | 100.00 | 100.00 | 104.00 | 104.00 |
| **Calculated nutrient composition** |  |  |  |  |
| GE (KJ/g) | 17.30 | 17.30 | 16.80 | 16.80 |
| CP (measured values) | 17.68 | 17.68 | 17.25 | 17.25 |
| CF (measured values) | 5.20 | 5.20 | 6.30 | 6.30 |
| CEE(measured values) | 6.20 | 6.20 | 6.10 | 6.10 |
| Lysine (measured values) | 0.60 | 0.60 | 0.70 | 0.70 |
| Methionine (measured values) | 0.15 | 0.15 | 0.15 | 0.15 |
| Threonine (measured values) | 0.61 | 0.61 | 0.69 | 0.69 |
| Ca (measured values) | 0.67 | 0.67 | 0.66 | 0.66 |
| Available P | 0.72 | 0.72 | 0.71 | 0.71 |

^1^ Premix provides the following ingredients (per kg) of the diet: VA, 9300 IU; VD, 3000 IU; VE, 15 IU; VK, 2.5 mg; VB_1_, 2 mg; VB_2_, 6.5 mg; VB_6_, 3 mg; VB_12_, 0.02 mg; nicotinicacid, 50 mg; pantothenicacid, 10 mg; folicacid, 0.9 mg; biotin, 0.15 mg; Cu, 8 mg; Fe, 80 mg; Mn, 90 mg; Zn, 60 mg; I, 0.35 mg

**Supplementary Table 2. Ingredients and nutrient compositions of diets (%) in the control (group C) and three experimental groups (B, F, and W) of Landes geese during the rearing stage (day 16 to 60).**

| **Ingredients** | **Group C** | **Group B** | **Group F** | **Group W** |
| --- | --- | --- | --- | --- |
| DDGC | 25.00 | 25.00 | 25.00 | 25.00 |
| Rice bran | 20.00 | 20.00 | 20.00 | 20.00 |
| Soybean meal | 2.64 | 2.64 | 2.64 | 2.64 |
| Brown rice mixture | 43.21 | 43.21 | 43.21 | 43.21 |
| Meat and bone powder | 2.50 | 2.50 | 2.50 | 2.50 |
| Fine stone powder | 0.59 | 0.59 | 0.59 | 0.59 |
| Paddy | 4.45 | 4.45 | 4.45 | 4.45 |
| BSG fermented | 0.00 | 0.00 | 4.00 | 4.00 |
| Methionine | 0.15 | 0.15 | 0.15 | 0.15 |
| Threonine | 0.10 | 0.10 | 0.10 | 0.10 |
| Lysine | 0.46 | 0.46 | 0.46 | 0.46 |
| NaCl | 0.20 | 0.20 | 0.20 | 0.20 |
| Choline chloride | 0.10 | 0.10 | 0.10 | 0.10 |
| NaHCO_3_ | 0.20 | 0.20 | 0.20 | 0.20 |
| Premix ^1^ | 0.40 | 0.40 | 0.40 | 0.40 |
| **Total** | 100.00 | 100.00 | 104.00 | 104.00 |
| **Calculated nutrient composition** |  |  |  |  |
| GE(KJ/g) | 16.80 | 16.80 | 16.71 | 16.71 |
| CP (measured values) | 15.69 | 15.69 | 15.47 | 15.47 |
| CF (measured values) | 4.60 | 4.60 | 5.20 | 5.20 |
| CEE(measured values) | 7.90 | 7.90 | 7.60 | 7.60 |
| Lysine (measured values) | 0.61 | 0.61 | 0.59 | 0.59 |
| Methionine (measured values) | 0.20 | 0.20 | 0.22 | 0.22 |
| Threonine (measured values) | 0.64 | 0.64 | 0.57 | 0.57 |
| Ca (measured values) | 0.478 | 0.478 | 0.45 | 0.45 |
| Available P | 0.97 | 0.97 | 0.94 | 0.94 |

^1^ Premix provides the following ingredients (per kg) of the diet: VA, 9600 IU; VD, 4800 IU; VE, 24 IU; VK, 2.4 mg; VB_1_, 2.5 mg; VB_2_, 10 mg; VB_6_, 5 mg; VB_12_ , 0.06 mg; nicotinicacid, 70 mg; pantothenicacid, 40 mg; folicacid, 2.4 mg; biotin, 0.25 mg; Cu, 8 mg; Fe, 80 mg; Mn, 90 mg; Zn, 60 mg; I, 0.35 mg

**Supplementary Table 3. Ingredients and nutrient compositions of diets (%) in the control (group C) and three experimental groups (B, F, and W) of Landes geese during the overfeeding stage (day 61 to 90).**

| **Ingredients** | **Group C** | **Group B** | **Group F** | **Group W** |
| --- | --- | --- | --- | --- |
| Corn | 88.64 | 88.64 | 88.64 | 88.64 |
| Brown rice mixture | 10.00 | 10.00 | 10.00 | 10.00 |
| BSG fermented | 0.00 | 4.00 | 0.00 | 4.00 |
| Soybean oil | 1.00 | 1.00 | 1.00 | 1.00 |
| NaCl | 0.10 | 0.10 | 0.10 | 0.10 |
| Enzyme complex | 0.05 | 0.05 | 0.05 | 0.05 |
| Phytase | 0.01 | 0.01 | 0.01 | 0.01 |
| Premix ^1^ | 0.20 | 0.20 | 0.20 | 0.20 |
| **Total** | 100.00 | 100.00 | 104.00 | 104.00 |
| **Calculated nutrient composition** |  |  |  |  |
| GE(KJ/g) | 15.80 | 15.90 | 15.80 | 15.90 |
| CP (measured values) | 8.28 | 8.48 | 8.28 | 8.48 |
| CF (measured values) | 1.30 | 1.90 | 1.30 | 1.90 |
| CEE(measured values) | 3.80 | 4.00 | 3.80 | 4.00 |
| Lysine (measured values) | 0.25 | 0.29 | 0.25 | 0.29 |
| Methionine (measured values) | 0.18 | 0.17 | 0.18 | 0.17 |
| Threonine (measured values) | 0.29 | 0.30 | 0.29 | 0.30 |
| Ca (measured values) | 0.0459 | 0.0516 | 0.0459 | 0.0516 |
| Available P | 0.27 | 0.28 | 0.27 | 0.28 |

^1^ Premix provides the following ingredients (per kg) of the diet: VA, 9600 IU; VD, 4800 IU; VE, 24 IU; VK, 2.4 mg; VB_1_, 2.5mg; VB_2_, 10 mg; VB_6_, 5 mg; VB_12_, 0.06 mg; nicotinicacid, 70 mg; pantothenicacid, 40 mg; folicacid, 2.4 mg; biotin, 0.25 mg; Cu, 8 mg; Fe, 80 mg; Mn, 90 mg; Zn, 60 mg; I, 0.35 mg

**Supplementary Table 4. Primers and their sequences used in the quantitative real-time PCR (qRT-PCR) analysis in this study. “F” and “R” at the end of each primer name indicate the forward and reverse primers, respectively.**

| **Primer** | **Sequence (5’-3’)** |
| --- | --- |
| β-actin-qF | CGAGCGGTTCAGGTGTCCA |
| β-actin-qR | CGTCGTATTCCTGCTTGCTG |
| CASQ2-qF | TGGACGAGCCTGTTCACATC |
| CASQ2-qR | TCCAGGAACTCAAAGCCGTC |
| SPR-qF | CTTCCTTGACCTCACCGACC |
| SPR-qR | CGCAGAGGGAGGAGATGTTC |
| PSPH-qF | ATGGCTCAAGACGGAGTCCA |
| PSPH-qR | TAGCTCTGCGGGTCATCTCT |
| HTR2C-qF | ATGGTGGTCTGATGGCTTGG |
| HTR2C-qR | TCACCGACATCCGAGAGACT |
| DGKI-qF | AAGGGTAAGACCCCTCGTGA |
| DGKI-qR | CCAAGGATCCTCTTTGCCGT |
| ELOVL7-qF | TTCGCCCCGTCTTGAGGAAG |
| ELOVL7-qR | CCAGCCTTCCAGTCTTGGAT |

**Supplementary Table 5. Summary of 16S rRNA sequencing data based on the cecal contents in the control (group C) and three experimental groups (B, F, and W) of Landes geese in 60 and 90 days.**

| **Sample** | **Valid read** | **Average length (base)** | **Total base** | **Q30 (%)** | **Q20 (%)** |
| --- | --- | --- | --- | --- | --- |
| C60_1 | 51,698 | 419.89 | 21,707,551 | 94.05 | 98.16 |
| C60_2 | 44,122 | 419.23 | 18,497,357 | 94.62 | 98.41 |
| C60_3 | 50,664 | 421.06 | 21,332,826 | 94.16 | 98.09 |
| C60_4 | 53,936 | 419.62 | 22,632,742 | 94.89 | 98.45 |
| C60_5 | 58,104 | 420.44 | 24,429,061 | 94.27 | 98.28 |
| C60_6 | 56,113 | 418.60 | 23,488,943 | 94.63 | 98.40 |
| C60_7 | 42,433 | 420.48 | 17,842,187 | 94.57 | 98.36 |
| C60_8 | 36,473 | 420.34 | 15,330,993 | 94.90 | 98.50 |
| B60_1 | 46,759 | 418.83 | 19,584,069 | 94.62 | 98.26 |
| B60_2 | 41,496 | 419.68 | 17,415,068 | 94.74 | 98.40 |
| B60_3 | 47,425 | 417.14 | 19,783,032 | 94.67 | 98.43 |
| B60_4 | 42,758 | 421.28 | 18,013,238 | 93.90 | 98.11 |
| B60_5 | 43,538 | 419.51 | 18,264,769 | 94.25 | 98.24 |
| B60_6 | 45,908 | 419.66 | 19,265,909 | 94.89 | 98.47 |
| B60_7 | 45,022 | 418.50 | 18,841,694 | 94.39 | 98.14 |
| B60_8 | 43,110 | 420.07 | 18,109,181 | 95.15 | 98.54 |
| F60_1 | 42,843 | 418.66 | 17,936,648 | 94.37 | 98.32 |
| F60_2 | 51,140 | 418.00 | 21,376,523 | 94.48 | 98.34 |
| F60_3 | 49,432 | 419.84 | 20,753,575 | 94.67 | 98.42 |
| F60_4 | 48,441 | 420.18 | 20,354,081 | 94.62 | 98.38 |
| F60_5 | 46,987 | 419.34 | 19,703,663 | 94.37 | 98.17 |
| F60_6 | 49,532 | 418.56 | 20,732,268 | 95.04 | 98.50 |
| F60_7 | 55,128 | 416.09 | 22,937,986 | 94.42 | 98.34 |
| F60_8 | 53,444 | 418.56 | 22,369,720 | 94.47 | 98.36 |
| W60_2 | 36,409 | 419.78 | 15,283,667 | 93.89 | 97.93 |
| W60_3 | 35,759 | 417.10 | 14,915,008 | 95.15 | 98.54 |
| W60_5 | 38,339 | 420.99 | 16,140,630 | 94.14 | 98.26 |
| W60_6 | 51,259 | 419.40 | 21,498,100 | 94.51 | 98.36 |
| W60_7 | 45,401 | 418.58 | 19,004,106 | 94.59 | 98.37 |
| W60_8 | 52,323 | 419.48 | 21,948,276 | 94.66 | 98.38 |
| C90_1 | 60,394 | 422.65 | 25,525,280 | 94.56 | 98.34 |
| C90_2 | 52,117 | 416.54 | 21,708,694 | 94.54 | 98.39 |
| C90_3 | 46,338 | 418.10 | 19,374,104 | 94.13 | 98.23 |
| C90_4 | 69,583 | 419.52 | 29,191,297 | 94.27 | 98.27 |
| C90_5 | 52,825 | 420.19 | 22,196,389 | 94.81 | 98.47 |
| C90_6 | 68,331 | 419.93 | 28,694,318 | 94.67 | 98.29 |
| C90_7 | 50,177 | 417.92 | 20,969,731 | 95.11 | 98.54 |
| C90_8 | 38,246 | 416.22 | 15,918,687 | 94.44 | 98.33 |
| B90_1 | 30,111 | 427.93 | 12,885,253 | 94.00 | 98.22 |
| B90_2 | 59,661 | 418.42 | 24,963,358 | 94.29 | 98.20 |
| B90_3 | 57,135 | 417.92 | 23,878,126 | 94.90 | 98.44 |
| B90_4 | 52,931 | 420.25 | 22,244,266 | 94.34 | 98.32 |
| B90_5 | 52,504 | 415.77 | 21,829,824 | 94.54 | 98.36 |
| B90_6 | 39,095 | 428.18 | 16,739,683 | 93.94 | 98.21 |
| B90_7 | 45,623 | 420.23 | 19,172,137 | 94.80 | 98.47 |
| B90_8 | 46,159 | 419.89 | 19,381,853 | 94.75 | 98.36 |
| F90_1 | 59,525 | 420.18 | 25,011,259 | 94.78 | 98.36 |
| F90_2 | 71,161 | 420.05 | 29,891,053 | 93.99 | 98.21 |
| F90_3 | 49,965 | 422.27 | 21,098,887 | 94.34 | 98.35 |
| F90_4 | 54,597 | 419.31 | 22,893,155 | 94.56 | 98.38 |
| F90_5 | 67,706 | 420.40 | 28,463,376 | 94.72 | 98.43 |
| F90_6 | 48,181 | 421.49 | 20,307,667 | 94.41 | 98.25 |
| F90_7 | 40,957 | 419.24 | 17,170,665 | 95.05 | 98.50 |
| F90_8 | 48,141 | 416.57 | 20,054,216 | 94.41 | 98.33 |
| W90_1 | 47,983 | 419.73 | 20,140,010 | 94.24 | 98.29 |
| W90_2 | 48,228 | 416.97 | 20,109,537 | 94.77 | 98.48 |
| W90_3 | 53,108 | 417.55 | 22,175,393 | 94.85 | 98.48 |
| W90_5 | 65,336 | 419.10 | 27,382,623 | 95.25 | 98.56 |
| W90_6 | 72,494 | 421.75 | 30,574,106 | 93.93 | 98.18 |
| W90_7 | 59,074 | 419.32 | 24,770,668 | 94.63 | 98.42 |
| W90_8 | 42,139 | 419.83 | 17,691,343 | 94.55 | 98.36 |

**Supplementary Table 6. Summary of sequencing data based on the liver transcriptomics analysis the control (group C) and three experimental groups (B, F, and W) of Landes geese in 60 and 90 days.**

| **Sample** | **Raw read** | **Raw base** | **Clean read** | **Clean base** | **Error rate (%)** | **Q20 (%)** | **Q30 (%)** |
| --- | --- | --- | --- | --- | --- | --- | --- |
| C90_1 | 50,161,638 | 7,574,407,338 | 49,310,310 | 7,264,238,570 | 0.0258 | 97.55 | 93.51 |
| C90_2 | 52,941,532 | 7,994,171,332 | 52,173,752 | 7,688,720,503 | 0.0256 | 97.66 | 93.72 |
| C90_3 | 52,494,646 | 7,926,691,546 | 51,772,174 | 7,636,095,022 | 0.0253 | 97.79 | 94.00 |
| C90_4 | 52,514,968 | 7,929,760,168 | 51,790,570 | 7,651,197,216 | 0.0255 | 97.70 | 93.80 |
| C90_5 | 41,899,696 | 6,326,854,096 | 41,376,196 | 6,144,186,938 | 0.0253 | 97.78 | 93.94 |
| C90_6 | 41,352,838 | 6,244,278,538 | 40,804,390 | 6,043,586,405 | 0.0254 | 97.72 | 93.88 |
| C90_7 | 53,487,646 | 8,076,634,546 | 52,713,640 | 7,770,086,971 | 0.0254 | 97.71 | 93.87 |
| C90_8 | 46,662,412 | 7,046,024,212 | 46,022,256 | 6,788,388,509 | 0.0256 | 97.68 | 93.76 |
| B90_1 | 46,625,782 | 7,040,493,082 | 46,035,294 | 6,803,056,954 | 0.0254 | 97.75 | 93.91 |
| B90_2 | 47,498,952 | 7,172,341,752 | 46,853,502 | 6,941,563,852 | 0.0254 | 97.75 | 93.96 |
| B90_3 | 45,336,576 | 6,845,822,976 | 44,703,938 | 6,605,113,719 | 0.0255 | 97.68 | 93.80 |
| B90_4 | 47,363,360 | 7,151,867,360 | 46,718,772 | 6,871,812,398 | 0.0254 | 97.73 | 93.91 |
| B90_5 | 48,802,632 | 7,369,197,432 | 48,150,686 | 7,114,235,748 | 0.0253 | 97.78 | 94.03 |
| B90_6 | 49,099,834 | 7,414,074,934 | 48,464,754 | 7,156,016,,673 | 0.0255 | 97.70 | 93.77 |
| B90_7 | 47,851,994 | 7,225,651,094 | 47,256,674 | 6,994,023,673 | 0.0254 | 97.72 | 93.86 |
| B90_8 | 48,442,886 | 7,314,875,786 | 47,843,350 | 7,088,113,456 | 0.0254 | 97.74 | 93.90 |
| F90_1 | 45,152,442 | 6,818,018,742 | 44,486,820 | 6,571,933,784 | 0.0256 | 97.67 | 93.74 |
| F90_2 | 46,756,874 | 7,060,287,974 | 46,140,138 | 6,879,969,273 | 0.0254 | 97.72 | 93.87 |
| F90_3 | 45,158,748 | 6,818,970,948 | 44,568,300 | 6,577,988,168 | 0.0264 | 97.37 | 92.88 |
| F90_4 | 42,165,372 | 6,366,971,172 | 41,587,526 | 6,154,714,579 | 0.0255 | 97.70 | 93.83 |
| F90_5 | 48,265,448 | 7,288,082,648 | 47,656,304 | 7,056,358,068 | 0.0255 | 97.73 | 93.84 |
| F90_6 | 49,813,034 | 7,521,768,134 | 49,161,182 | 7,289,493,266 | 0.0257 | 97.64 | 93.62 |
| F90_7 | 43,480,988 | 6,565,629,188 | 42,893,050 | 6,332,053,157 | 0.0253 | 97.76 | 93.96 |
| F90_8 | 47,513,966 | 7,174,608,866 | 46,851,040 | 6,932,522,499 | 0.0255 | 97.68 | 93.79 |
| W90_1 | 56,262,704 | 8,495,668,304 | 55,563,130 | 8,170,828,288 | 0.0253 | 97.77 | 93.95 |
| W90_2 | 51,610,212 | 7,793,142,012 | 50,918,930 | 7,549,845,199 | 0.0255 | 97.70 | 93.78 |
| W90_3 | 46,161,756 | 6,970,425,156 | 45,527,500 | 6,748,451,319 | 0.0255 | 97.68 | 93.78 |
| W90_4 | 47,675,140 | 7,198,946,140 | 47,114,968 | 7,002,436,829 | 0.0253 | 97.78 | 93.93 |
| W90_5 | 50,451,972 | 7,618,247,772 | 49,782,020 | 7,337,911,280 | 0.0255 | 97.70 | 93.84 |
| W90_6 | 48,696,362 | 7,353,150,662 | 48,031,992 | 7,109,635,332 | 0.0257 | 97.60 | 93.61 |
| W90_7 | 58,431,306 | 8,823,127,206 | 57,639,040 | 8,475,894,167 | 0.0255 | 97.69 | 93.78 |
| W90_8 | 47,492,634 | 7,171,387,734 | 46,945,280 | 6,955,824,467 | 0.0254 | 97.75 | 93.89 |
